# Enhanced photoluminescence of strongly coupled single molecule-plasmonic nanocavity: analysis of spectral modifications using nonlocal response theory

**DOI:** 10.1515/nanoph-2024-0580

**Published:** 2025-02-17

**Authors:** Yoshitsugu Tomoshige, Mamoru Tamura, Tomohiro Yokoyama, Hajime Ishihara

**Affiliations:** Department of Materials Engineering Science, 13013 Osaka University, Osaka, Japan; RILACS, Osaka Metropolitan University, Osaka, Japan; Ritsumeikan Semiconductor Application Research Center (RISA), Ritsumeikan University, Kusatsu, Shiga, Japan

**Keywords:** plasmonic nanocavity, single molecule, photoluminescence, strong coupling, Rabi splitting

## Abstract

Plasmonic nanocavities with highly localized fields in their nanogaps significantly enhance light–matter interactions at the nanoscale, surpassing the diffraction limit. Strong coupling between a plasmonic nanocavity and a molecule forms hybrid upper and lower branch states, resulting in Rabi splitting (RS) in optical spectra. However, scattering and absorption spectra often fail to unambiguously distinguish whether the double peaks arise from energy transparency or RS. In contrast, photoluminescence (PL) clearly reveals the quantum state of a molecule coupled with a plasmon by filtering out background fields. This paper presents a theoretical framework based on nonlocal response theory to calculate the PL of a single molecule coupled with arbitrary metallic nanostructures. Our theory provides an analytical approach to design the spatial arrangement of metallic nanostructures and molecular orbitals and to calculate the PL in strongly coupled systems, addressing limitations in previous studies. Using this framework, we investigated a coupled system comprising a gold nanoplate dimer and a planar porphyrin tape. By modifying porphyrin units to modulate coupling strength, we explored the molecular quantum state coupled with the nanocavity through PL analysis. We elucidated the spectral features of absorption, excitation, and PL in weak and strong coupling regimes and evaluated the dependence of coupling strength on the molecular position and orientation within the nanogap. Our results demonstrate that the quantum state of a molecule in an optically forbidden transition can be excited by the highly localized field in the nanogap. This work advances the fundamental understanding of light–matter interactions at the nanoscale and provides a foundation for the development of future nanophotonic devices.

## 1 Introduction

Localized surface plasmon resonance (LSPR) enhances light–matter interactions at the nanoscale by generating localized light beyond the diffraction limit. Plasmonic nanocavities, such as metallic bowtie and tip-substrate structures, concentrate enhanced fields in nanogaps through LSPR [1]. These structures have diverse applications, including significant fluorescence enhancement [2], [3], [4], nanomaterial manipulation via optical gradient forces [5], [6], single-molecule spectroscopic imaging using scanning probe microscopy [7], [8], [9], [10], and plasmonic catalysis for chemical reaction control [11].

When the coupling strength between nanomaterials and plasmons surpasses the relaxation rates of both, the system enters a strong coupling regime [12], [13]. In this regime, the exciton state of the nanomaterial and plasmon hybridizes into upper and lower branch states, resulting in Rabi splitting (RS) in the optical spectrum [14], [15], [16], [17]. Strong coupling between plasmonic nanocavities and a few or single emitters has even been demonstrated at room temperature [18], [19], [20], [21]. Such systems are promising for applications like photochemical reaction control [22], [23], [24], single-emitter lasers [25], enhancing the performance of organic light-emitting diodes (OLEDs) [26], and plasmonic sensing [27], [28]. Advancing these applications requires a fundamental understanding and effective designs for strong coupling systems involving plasmonic nanocavities and nanomaterials.

For a single molecule as an emitter, the design flexibility significantly increases due to the degrees of freedom in molecular structures, cavity geometries, and their interplay. This flexibility is a crucial advantage for exploring novel functionalities in cavity-emitter systems. Among experimental techniques, photoluminescence (PL) is particularly advantageous as it provides exclusive information on individual molecular emitters. In contrast, absorption and scattering spectra, while informative about both emitters and metallic nanostructures, often introduce ambiguities in observing RS due to plasmonic signals that mimic RS-like double peaks via energy transparency (ET) effects [29], [30], [31], [32]. PL, however, filters out plasmonic scattering fields and redshifts the emission, offering direct insights into quantum states of molecules coupled with plasmons [32], [33].

Significant progress has been made in theoretical studies on plasmon-single molecule systems [34], [35], [36], yet challenges in PL calculations persist. Previous models often parameterize the coupling strength (*g*) without considering the geometric characteristics of metallic nanostructures or spatial molecular orbital distributions [37]. Some studies use Green’s functions to model metallic structures but simplify emitters as point dipoles [38]. Others incorporate molecular orbitals and metallic geometries but fix the Green’s function frequency to the molecular resonance, limiting their applicability in strong coupling scenarios [39]. Thus, developing a comprehensive theoretical framework for PL under strong coupling conditions is essential. This framework must address large shifts in coupled states due to self-consistent light-molecule interactions and explicitly consider molecular geometries leading to nonlocal optical responses.

To address these challenges, we propose a theoretical framework for PL based on nonlocal response theory. This framework calculates PL spectra in systems where a single molecule couples to metallic nanostructures. Our approach self-consistently describes polarization by incorporating molecular orbital distributions and electric fields through Green’s functions. This method, which formulates PL as a function of emission frequency without approximation, enables PL analyses in strong coupling regimes. We demonstrate its utility by applying it to a coupled system of a metallic nanoplate dimer and a single molecule. Comparing absorption, excitation, and PL spectra, we show that the PL spectrum of an excited molecule, including optically forbidden transitions, can be captured through the localized field in the nanogap.

## 2 Theoretical framework

**Figure 1:**
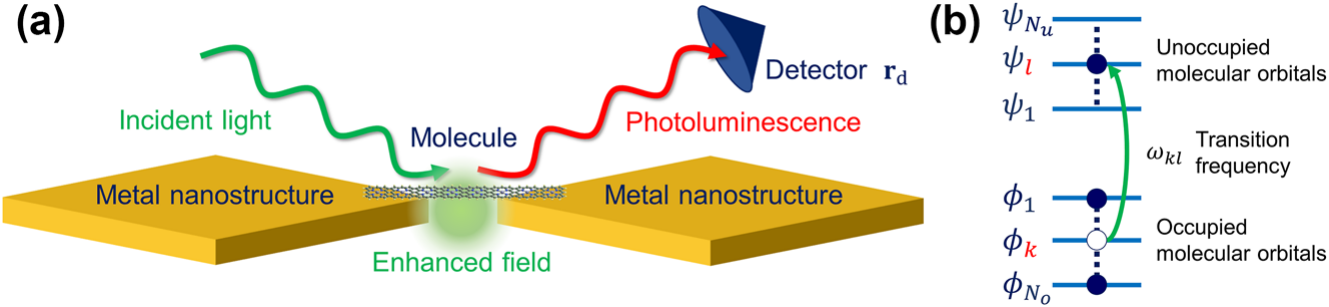
Schematic of theoretical model. (a) Schematic representation of the plasmonic nanocavity-molecule coupled system. (b) Energy level diagram of a single molecule.

Figure 1(a) shows a schematic representation of the plasmonic nanocavity-molecule coupled system, while Figure 1(b) illustrates a schematic energy level diagram of a single molecule. The incident light excites the molecule, causing an electron to transition from an occupied molecular orbital (*ϕ*_*k*_) to an unoccupied orbital (*ψ*_*l*_), as depicted in Figure 1(b). In realistic scenarios, vibronic states contribute to optical responses. For instance, the PL peaks of the different vibrational modes of a single molecule coupled with a plasmonic tip can be obtained through relaxation processes from the selectively excited electronic state, thereby enabling the spectroscopic distinction of closely related molecules owing to the high energy resolution [33]. However, in this study, we omit them to focus on the differences between various types of spectra in strong coupling systems, avoiding nonessential and complex spectral structures. The molecule, coupled with the localized surface plasmon (LSP) in the nanogap, emits PL observed by the detector in the far-field regime. We assume that PL is emitted from the excited level due to faster radiative relaxation compared to interlevel relaxation, similar to the ultrafast fluorescence of molecules coupled with surface plasmons [40]. The PL is evaluated using the following theoretical framework. The Hamiltonian of the plasmonic nanocavity-molecule coupled system is described as:

(1) $$\hat{H}={\hat{H}}_{\text{mol}}+{\hat{H}}_{\text{rad}}+{\hat{H}}_{\text{int}}.$$

The Hamiltonian of the single molecule is described as ${\hat{H}}_{\text{mol}}=\sum_{k,l}\hbar \omega_{kl}{\hat{b}}_{kl}^{†}{\hat{b}}_{kl},$ where *ω*_*kl*_ is the transition frequency between the molecule orbital *ϕ*_*k*_ and *ψ*_*l*_, and ${\hat{b}}_{kl}\;\left({\hat{b}}_{kl}^{†}\right)$ denote bosonic annihilation (creation) operators of the one-electron excitation between *ϕ*_*k*_ and *ψ*_*l*_. In Eq. (1), the second term represents the Hamiltonian of the radiation field, ${\hat{H}}_{\text{rad}}=\sum_{\eta }\hbar \Omega_{\eta }{\hat{a}}_{\eta }^{†}{\hat{a}}_{\eta },$ where ${\hat{a}}_{\eta }\;\left({\hat{a}}_{\eta }^{†}\right)$ are the bosonic annihilation (creation) operators for the *η*th photon with eigenfrequency Ω_*η*_. The third term in Eq. (1) describes the Hamiltonian of the interaction between the molecule and the electric field:

(2) $${\hat{H}}_{\text{int}}=-\int dr\;{\hat{P}}_{\text{mol}}\left(r\right)\cdot \hat{E}\left(r\right).$$

${\hat{P}}_{\text{mol}}\left(r\right)$ is the molecular polarization operator, expressed as ${\hat{P}}_{\text{mol}}\left(r\right)=\sum_{k,l}\left[P_{kl}\left(r\right){\hat{b}}_{kl}+P_{kl}^{*}\left(r\right){\hat{b}}_{kl}^{†}\right],$ and $P_{kl}\left(r\right)$ represents the transition dipole density. The distribution of the transition dipole density is determined using the local dipole moment in a small unit volume *V*_*j*_ around the position **r**_*j*_, expressed as: $\delta \mu_{kl}^{j}=\frac{ie}{\omega_{kl}}\int_{V_{j}}dr\;j_{kl}\left(r\right)$ [41], where **j**_*kl*_(**r**) is the transition current density, described as:

(3) $$j_{kl}\left(r\right)=-\frac{i\hbar }{2m_{e}}\left[\psi_{l}^{*}\left(r\right)\nabla \phi_{k}\left(r\right)-\phi_{k}^{*}\left(r\right)\nabla \psi_{l}\left(r\right)\right],$$

with *m*_*e*_ being the mass of the electron [42].

From master equation [43], the motion equation of the polarization is derived as:

(4) $$\begin{array}{ll} \frac{\partial }{\partial t}\;\left\langle {\hat{b}}_{kl}\left(t\right)\right\rangle & =-\left(i\omega_{kl}+\frac{\gamma }{2}+\frac{\Gamma }{2}\right)\left\langle {\hat{b}}_{kl}\left(t\right)\right\rangle \\ & \;+\frac{i}{\hbar }\int dr\;P_{kl}^{*}\left(r\right)\cdot \left\langle {\hat{E}}_{out}\left(r,t\right)\right\rangle , \end{array}$$

where $\left\langle {\hat{b}}_{kl}\left(t\right)\right\rangle$ is defined as $\left\langle {\hat{b}}_{kl}\left(t\right)\right\rangle \equiv \mathrm{Tr}\left[\hat{\rho }\left(t\right){\hat{b}}_{kl}\right]$ , and *γ* and Γ represent the nonradiative damping and the dephasing terms, respectively. Applying the Fourier transform to Eq. (4) and substituting the result into the expression for molecular polarization under the rotating wave approximation, we obtain

(5) $$〈{\hat{P}}_{\text{mol}}\left(r,\omega \right)〉=\varepsilon_{0}\int dr^{\prime }\chi_{\text{mol}}\left(r,r^{\prime },\omega \right)〈{\hat{E}}_{out}\left(r^{\prime },\omega \right)〉,$$

where *χ*_mol_(**r**, **r**′, *ω*) is the nonlocal susceptibility, expressed as:

(6) $$\chi_{\text{mol}}\left(r,r^{\prime },\omega \right)=\frac{1}{\varepsilon_{0}}\underset{k,l}{\sum }\frac{P_{kl}\left(r\right)P_{kl}^{*}\left(r^{\prime }\right)}{\hbar \omega_{kl}-\hbar \omega -i\hbar \gamma /2-i\hbar \Gamma /2}.$$

The nonlocality in the molecular polarization becomes particularly significant in optically forbidden transitions involving several nodes within the molecule [44].

Using input–output theory and Fourier transformation, Maxwell’s electric field can be expressed as:

(7) $$\begin{array}{ll} \left\langle {\hat{E}}_{out}\left(r,\omega \right)\right\rangle & =\left\langle {\hat{E}}_{in}\left(r,\omega \right)\right\rangle \\ & \;+\int dr^{\prime }G\left(r,r^{\prime },\omega \right)\left\langle {\hat{P}}_{\text{mol}}\left(r^{\prime },\omega \right)\right\rangle , \end{array}$$

where $\left\langle {\hat{E}}_{in}\left(r,\omega \right)\right\rangle$ represents the incident field, and **G**(**r**, **r**′, *ω*) is the Green’s function, which renormalizes arbitrary metallic structures. The Green’s function encompasses all eigenmodes of the electric field described in ${\hat{H}}_{\text{rad}}$ . The detailed expression can be found in S1 of the Supplementary Material. The renormalized Green’s function is defined as [45], [46]

(8) $$\begin{array}{ll} G\left(r,r^{\prime },\omega \right) & =G_{0}\left(r,r^{\prime },\omega \right)\\ & \;+\int dr^{\prime \prime }G_{0}\left(r,r^{\prime \prime },\omega \right)\varepsilon_{0}\chi_{\text{met}}\left(r^{\prime \prime },\omega \right)G\left(r^{\prime \prime },r^{\prime },\omega \right). \end{array}$$

Here, **G**_0_(**r**, **r**′, *ω*) represents the free-space Green’s function, and *χ*_met_(**r**, *ω*) denotes metallic susceptibility. In this framework, the nonlocal effect of metal [47], [48] is neglected because the separation between the metallic surface and molecules is sufficiently large to prevent wavefunction mixing [8]. The renormalized Green’s function was calculated using discrete dipole approximation [49], [50]. In this framework, the incident field is treated as classical continuous light, expressed as: $\left\langle {\hat{E}}_{in}\left(r,\omega \right)\right\rangle =E_{\text{bg}}\left(r,\omega \right)\delta \left(\omega -\omega_{in}\right),$ where $E_{\text{bg}}\left(r,\omega \right)$ represents the background field from the metallic structure without the molecule, and *ω*_in_ denotes the frequency of the incident light.

The molecular polarization and electric field were determined through the self-consistent solution of Eqs. (5) and (7), which collectively describe the coupling between the molecule and the nanocavity.

The luminescence in a steady-state system is related to the power spectrum, which is calculated using the Fourier transformation of the autocorrelation function, as described by the Wiener–Khintchine theorem [51], [52]. The autocorrelation function of the output field is given by: $\left\langle \Delta {\hat{E}}_{out}^{†}\left(r,t\right)\Delta {\hat{E}}_{out}\left(r,t+\tau \right)\right\rangle =\left\langle {\hat{E}}_{out}^{†}\left(r,t\right){\hat{E}}_{out}\left(r,t+\tau \right)\right\rangle -\left\langle {\hat{E}}_{out}^{†}\left(r,t\right)\right\rangle \left\langle {\hat{E}}_{out}\left(r,t+\tau \right)\right\rangle$ . The PL spectrum observed at the detector position **r**_d_ is calculated as:

(9) $$\begin{array}{ll} & S_{\text{inc}}\left(r_{\text{d}},\omega_{out}\right)=\frac{1}{\pi }R\left[\overset{\infty }{\underset{0}{\int }}d\tau \;{\text{e}}^{\text{i}\omega_{out}\tau }\left\langle \Delta {\hat{E}}_{out}^{†}\left(r_{\text{d}},t\right)\Delta {\hat{E}}_{out}\left(r_{\text{d}},t+\tau \right)\right\rangle \right]. \end{array}$$

Using Maxwell’s electric field, as expressed in Eq. (7), we derived the following formula for the PL spectrum:

(10) $$\begin{array}{ll} S_{\text{inc}}\left(r_{\text{d}},\omega_{out}\right) & =\frac{2}{\pi }\text{Re}\left[\underset{k,l,k^{\prime },l^{\prime }}{\sum }F_{kl}^{*}\left(r_{\text{d}},\omega_{out}\right)\right.\\ & \left.\;\cdot F_{k^{\prime }l^{\prime }}\left(r_{\text{d}},\omega_{out}\right)\Delta {\bar{B}}_{kl,k^{\prime }l^{\prime }}\left(\omega_{out}\right)\right]. \end{array}$$

The electric field propagating to the detector position **r**_d_ is expressed as:

(11) $$F_{kl}\left(r_{\text{d}},\omega \right)\equiv \int dr^{\prime }G\left(r_{\text{d}},r^{\prime },\omega \right)P_{kl}\left(r^{\prime }\right).$$

$\Delta {\bar{B}}_{kl,k^{\prime }l^{\prime }}\left(\omega_{out}\right)$ is calculated by the Laplace transformation of the autocorrelation function $\Delta {\bar{B}}_{kl,k^{\prime }l^{\prime }}\left(\tau \right)$ with respect to delay time *τ*, using quantum regression theorem [53]. The detailed calculation is given by S1 of Supplementary Material. The autocorrelation function $\Delta {\bar{B}}_{kl,k^{\prime }l^{\prime }}\left(\tau \right)$ is defined as: $\Delta {\bar{B}}_{kl,k^{\prime }l^{\prime }}\left(\tau \right)=\left\langle {\hat{b}}_{kl}^{†}\left(t\right){\hat{b}}_{k^{\prime }l^{\prime }}\left(t+\tau \right)\right\rangle -\left\langle {\hat{b}}_{kl}^{†}\left(t\right)\right\rangle \left\langle {\hat{b}}_{k^{\prime }l^{\prime }}\left(t+\tau \right)\right\rangle$ . It is important to note that Eq. (10) is explicitly expressed in terms of the PL frequency, enabling the analysis of PL in a strong coupling regime. This theory can be extended to similar formulations for multiple molecules by incorporating molecular indices. The further information about theoretical equations are presented in Section S1 of the Supplementary Material.

## 3 Results and discussion

In our calculation model, we focused on the coupling between a gold nanoplate dimer and a planar porphyrin tape, each comprising multiple porphyrin units [54], [55]. Understanding the coupling between a linear molecule and a small gap could enable applications in molecular junctions [56], [57]. Experimentally, samples with the desired molecular configuration can be prepared and identified using methods similar to those described in Ref. [58]. We selected porphyrin tapes containing two, four, and eight units. As the number of units increases, the transition dipole moment of the entire porphyrin tape becomes stronger, enhancing the coupling with the nanocavity. The nanoplate size was adjusted to align the plasmon resonance with the molecular resonance for each tape type. Initially, we analyzed the coupling state between the nanoplate dimer and one or a few two-unit porphyrin tapes. Subsequently, we investigated the strong coupling of four- and eight-unit tapes and, finally, addressed the coupling of optically forbidden transitions for a single eight-unit porphyrin tape. These analyses reveal how the transition from weak to strong coupling manifests differently in PL, excitation, and absorption spectra.

### 3.1 Array of two-unit porphyrin tape

**Figure 2:**
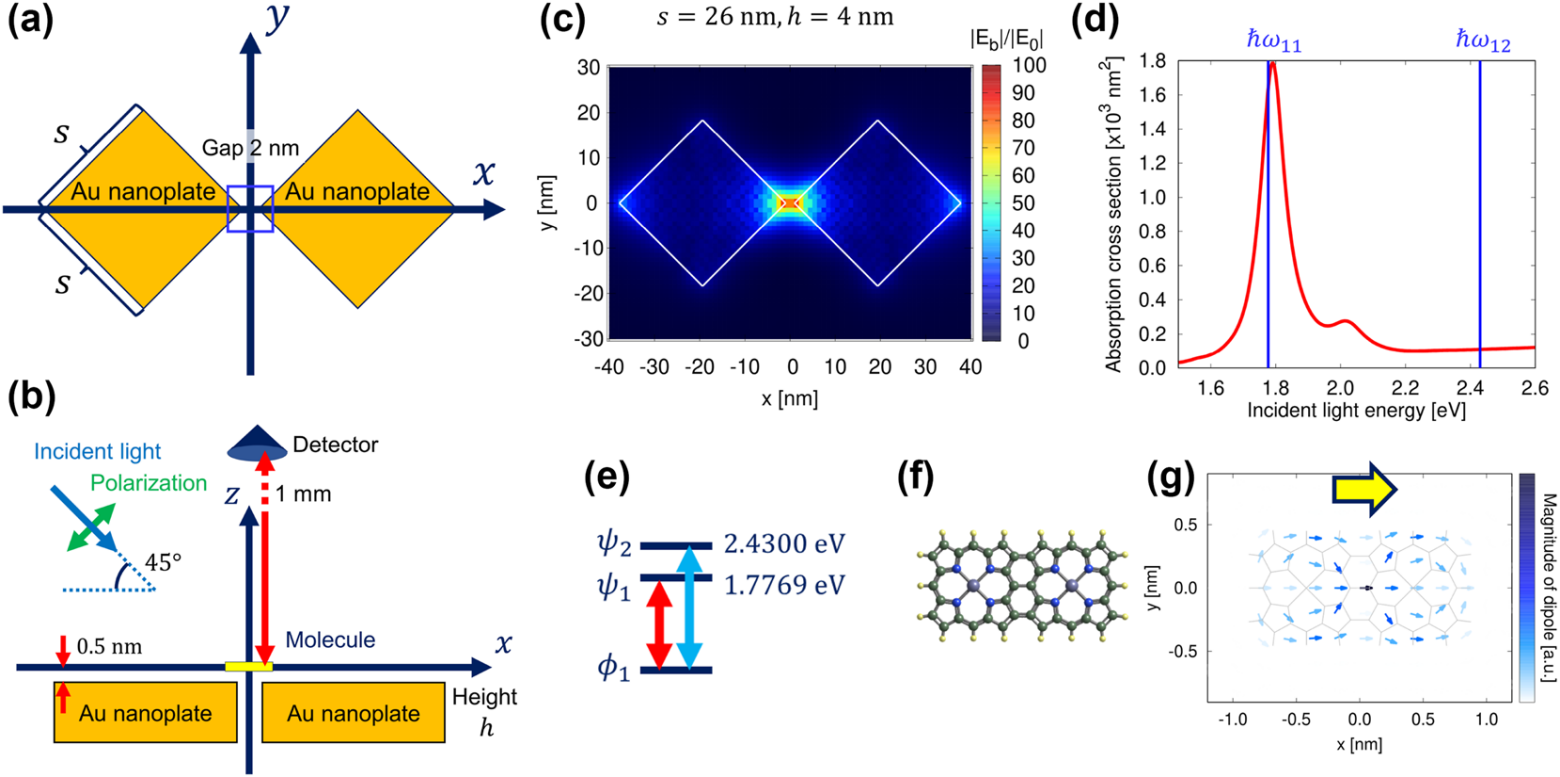
Schematic of calculation model. (a) Top view and (b) side view of the gold nanoplate dimer, showing the detector position, incident p-polarized light, and the molecule located above the nanogap. The blue-outlined square in (a) marks the area where the molecule is positioned near the nanogap. The side length and height of the nanoplates are denoted as *s* and *h*, respectively. The molecule is placed 0.5 nm above the nanoplate, with a spacer assumed to prevent wavefunction overlap between the metal and molecule, as well as quenching effects. (c) Electric field distribution at *z* = 0 without the molecule for *s* = 26 nm and *h* = 4 nm; white lines outline the nanoplate structure. (d) Absorption spectrum of the metallic nanostructure without molecules, with blue lines indicating the molecular resonance energy. (e) Energy level diagram and (f) molecular structure of the conjugated porphyrin. (g) Transition dipole distribution in the first excited state, showing the transition between *ϕ*_1_ and *ψ*_1_, with yellow arrows indicating the net transition dipole moment within the molecule.

Figure 2(a) and (b) illustrates the top and side views of our calculation model, respectively, showing the geometric configuration of the gold nanoplate dimer, the molecule, the detector position, and the incident light. We assumed a gap size of 2 nm, which can be fabricated using electron beam lithography [59]. For gold susceptibility, we employed parameters from the Drude and critical points model [60]. The surrounding medium was set as vacuum, which remains consistent for all subsequent calculation models. The side length and height of the nanoplates were defined as *s* and *h*, respectively, to modulate the nanoplate size and align the plasmon resonance with the first molecular resonance. The incident light had an elevation angle of 45° relative to the nanoplate, and its polarization, depicted in Figure 2(b), included a component parallel to the alignment of the nanoplates. The incident intensity was set to 1 μW/mm^2^. The molecule was positioned 0.5 nm above the nanoplate, with a spacer assumed to prevent wavefunction overlap between the metal and molecule, as well as quenching effects. (Note that our results inherently include quenching effects caused by dipole interactions, which depend on the molecule-metal distance through self-consistent calculations.) Such spacers can be achieved by growing a thin NaCl layer [61], [62] or by doping the molecule into a thin polymer [2]. This spacer length is deemed sufficient, as PL enhancement has been demonstrated even at metallic tip-molecule distances as small as approximately 370 pm, as reported in [8]. Figure 2(c) shows the electric field distribution in the metallic nanostructure without the molecule, for *s* = 26 nm, *h* = 4 nm, and *ℏω*_in_ = 1.7769 eV, corresponding to the molecular resonance of the first excited state. The enhanced field is localized within the nanogap. The profile of the electric field along the *z* direction at *x*, *y* = 0 nm is provided in S2 of the Supplementary Material. Figure 2(d) presents the absorption spectrum of the metallic nanoplates without the molecule, calculated for *s* = 26 nm and *h* = 4 nm, using the absorption cross-sectional formula [31], [46].

First, we examined a porphyrin tape with two units. The blue lines in Figure 2(d) indicate the transition energies of the porphyrin tape, as depicted in the energy diagram in Figure 2(e). Several plasmon modes are observed in Figure 2(d), with the plasmon mode exhibiting a large peak near the molecular resonance contributing most significantly to the coupling with the molecule. The peak near 2.0 eV in Figure 2(d) corresponds to a higher-order plasmonic mode, which is also evident in the absorption spectrum of the subsequent calculation model. The electric field distributions for these higher-order plasmonic modes are provided in S3 of the Supplementary Material. The molecular orbitals and eigenenergies were calculated using the GAMESS quantum chemical calculation software [63]. The relaxation parameters of the molecule were set to *ℏγ* = 5 meV and *ℏ*Γ = 5 meV. As shown in Figure 2(d), the molecular resonance of the first excited state, corresponding to the transition between the molecular orbitals *ϕ*_1_ and *ψ*_1_, lies within the plasmon resonance of the gold nanoplate dimer. In contrast, the molecular resonance of the second excited state, corresponding to the transition between *ϕ*_1_ and *ψ*_2_, is outside the plasmon resonance. Figure 2(f) and (g) illustrates the molecular structure and the transition dipole distribution in the first excited state of the porphyrin tape. The net dipole moment is oriented in the *x*-direction, as indicated by the yellow arrows in Figure 2(g).

**Figure 3:**
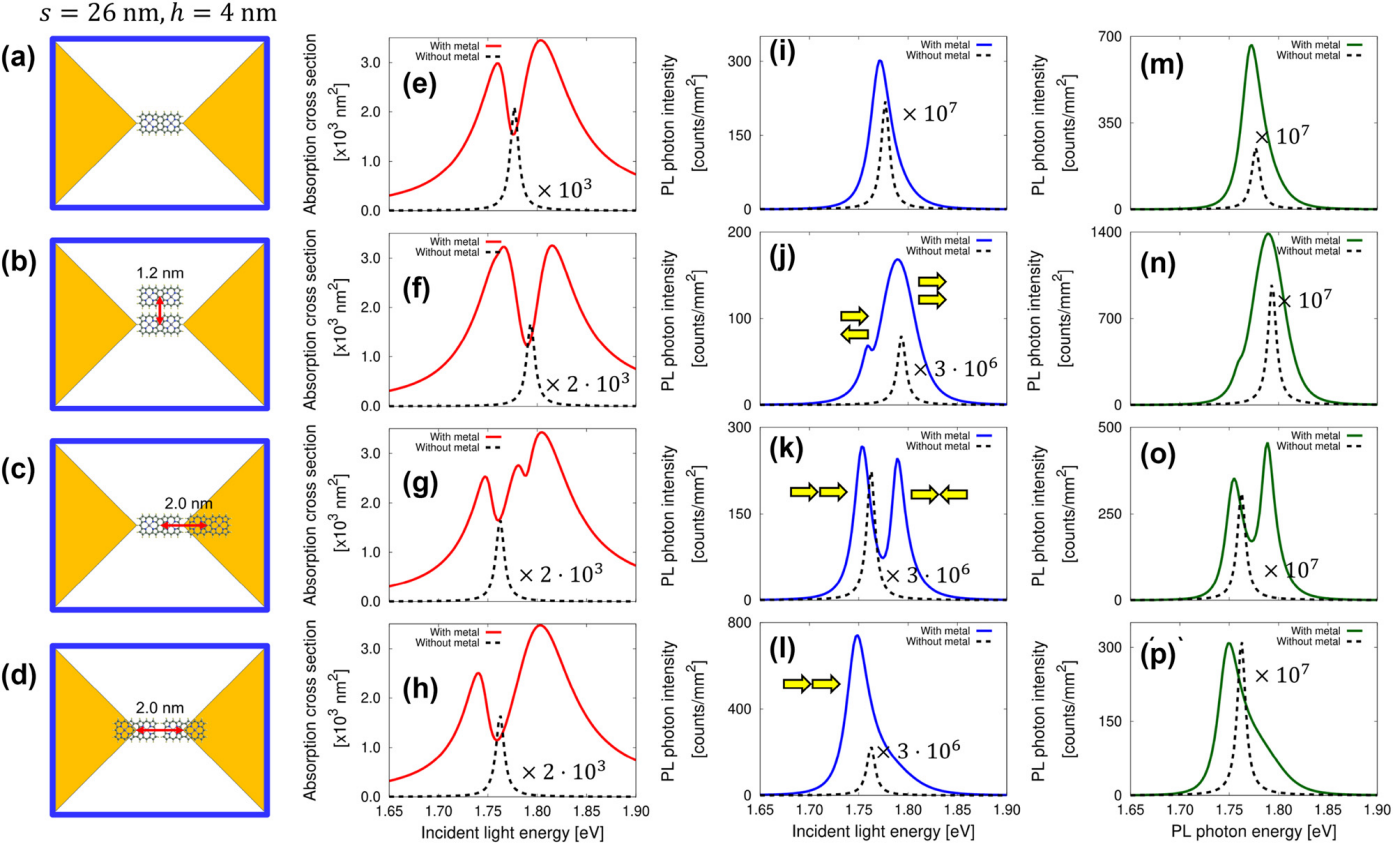
Spectra for two-unit porphyrin tapes. (a) Schematic representation of a single porphyrin tape positioned at the center of the nanogap, (b) two porphyrin tape arrays in parallel configuration, (c) row configuration displaced from the center, and (d) row configuration at the center of the nanogap. (e–h) Absorption spectra of the coupled system (red line), (i–l) excitation spectra (blue line), and (m–p) PL spectra (green line) for configurations (a–d). In each spectrum, the dashed black line represents the spectrum without the metallic nanostructure. For (j), (k), and (l), yellow arrows indicate the molecular polarization modes.

As illustrated in Figure 3, the schematic models depict: (a) a single porphyrin tape positioned at the center of the nanogap, (b) an array of two porphyrin tapes in parallel configuration, (c) a row configuration displaced from the center, and (d) a row configuration at the center of the nanogap. Figure 3(e)–(h) presents the absorption spectra of the coupled system (red line) and the absorption spectra of the molecule without the metallic nanostructure (dashed black line) for configurations (a), (b), (c), and (d), respectively. The absorption spectra of the coupled system represent the combined absorption of the metallic nanostructure and the molecule. Figure 3(i)–(l) displays the excitation spectra, obtained using the PL intensity at *ℏω*_out_ = 1.7169 eV, with the metallic nanostructure (blue line) and without it (dashed black line) for configurations (a), (b), (c), and (d), respectively. The output energy is chosen to be shifted 60 meV from the molecular resonance, under the assumption that PL with lower energy than the excited state level passes through the filter. Similarly, for subsequent models, the output energy is selected at a lower level than the molecular resonance. The choice of this energy allows for a certain degree of flexibility as the spectral structure remains unaltered by the selection of the output energy. Compared with the PL intensity in vacuum, a PL enhancement of up to approximately 10^7^ times is observed. For the configuration shown in Figure 3(a), the absorption spectrum exhibits a dip at the peak energy of the excitation spectrum, indicating ET [30], [64], while the excitation spectrum reveals molecular absorption that is otherwise obscured by substantial metal absorption. The yellow arrows in the inset of Figure 3(j)–(l) indicate the molecular polarization modes corresponding to each peak. The array of molecules exhibits parallel and antiparallel polarization modes for the configuration in Figure 3(b), and bonding and antibonding modes for the configurations in Figure 3(c) and (d), due to molecular interactions. The excitation spectrum reveals not only the peak resulting from the parallel mode but also the antiparallel mode, due to the displacement of the molecular array from the center nanogap. This explicit signal is not apparent in the absorption spectrum. Similarly, for the configuration shown in Figure 3(c), the row array of molecules exhibits bonding and antibonding modes, with two dips in the absorption spectrum and two peaks in the excitation spectrum reflecting these states. For the configuration shown in Figure 3(d), the dip and peak resulting from the antibonding mode are absent in both spectra due to the high symmetry of the molecular array and nanogap. The coupling becomes substantial, leading to broadened dips that result in an RS-like structure in the absorption spectrum. The excitation spectrum elucidates the explicit quantum states of the molecules coupled with the nanocavity, particularly in the intermediate coupling regime, where the coupled system has not yet attained the strong coupling regime.

Additionally, the PL spectra are shown in Figure 3(m)–(p) for the configurations in Figure 3(a)–(d), respectively. The spectra are presented with the metallic nanostructure (green line) and without it (dashed black line), where the incident light energy is set at *ℏω*_in_ = 1.8369 eV. The incident light energy is selected to be higher level the molecular resonance energy, which is similar in subsequent models. The PL spectral structure remains unaffected by the choice of incident light energy. While the PL spectra closely match the peak energies, minor discrepancies are observed. The differences between the excitation and PL spectra become prominent in the strong coupling regime, which will be discussed in detail in Sections 3.2 and 3.3.

### 3.2 A single four-unit porphyrin tape

**Figure 4:**
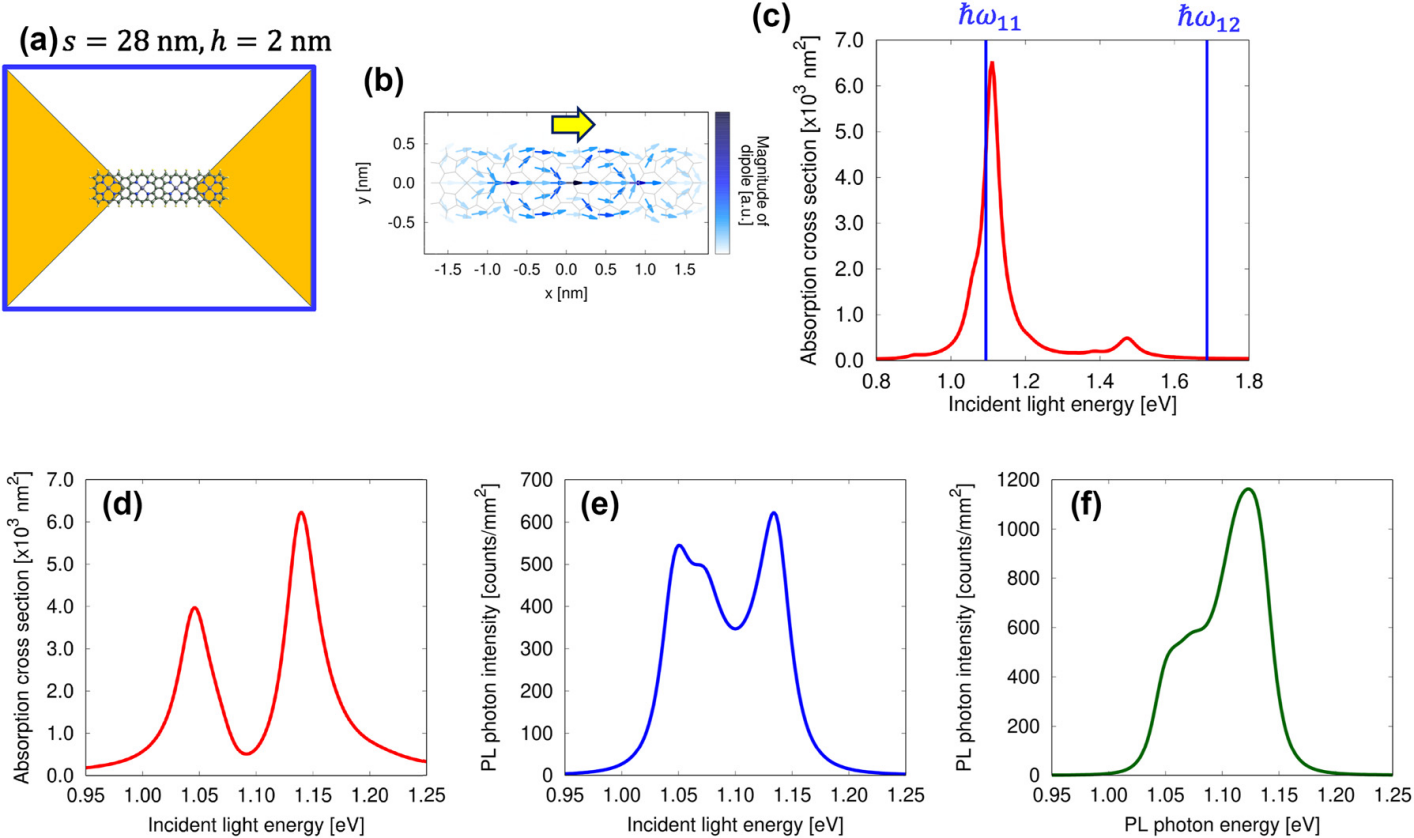
Spectra for a single four-unit porphyrin tape. (a) Schematic model of a porphyrin tape with four units positioned at the center of the nanogap between the nanoplates, with *s* = 28 nm and *h* = 2 nm. (b) Distribution of the transition dipole in the first excited state, with a yellow arrow indicating the net direction of the transition dipole moment. (c) Absorption spectrum of the nanoplate dimer for *s* = 28 nm and *h* = 2 nm, with blue lines representing the resonance energy of the porphyrin tape with four units. (d) Absorption spectrum of the coupled system, (e) excitation spectrum, and (f) PL spectrum.

In this section, we examine the strong coupling state utilizing porphyrin tapes with four and eight units. Specifically, we investigate the coupling state of the tape with eight units in detail as a function of molecular position and rotation angle. Figure 4(a) depicts a porphyrin tape with four units positioned at the center of the nanogap between nanoplates, with *s* = 28 nm and *h* = 2 nm. Figure 4(b) illustrates the transition dipole distribution in the first excited state of the porphyrin tape, with a net dipole moment in the *x*-direction, as indicated by yellow arrows. Figure 4(c) presents the absorption spectrum of the metallic nanoplates without the molecule, with blue lines indicating the molecular resonance energy of the porphyrin tape with four units. Figure 4(d) and (e) displays the absorption spectrum of the coupled system and the excitation spectrum derived from the PL intensity at *ℏω*_out_ = 1.0139 eV, respectively. These spectra exhibit splitting peaks, indicating that this coupled system is in a strong coupling state. As shown in Figure 4(e), a central peak appears between the lower and upper branches, originating from weak coupling with another plasmon mode. Figure 4(f) shows the PL spectrum obtained using incident light with *ℏω*_in_ = 1.1939 eV. In this case, the RS is obscured due to the plasmon linewidth. To obtain a distinct RS in the excitation spectrum, as demonstrated later for the eight-unit porphyrin tape, interaction with a single plasmon mode and the exciton mode of the molecule is required. These results demonstrate that the porphyrin tape with four units possesses a sufficiently large transition dipole moment to form a strong coupling state.

### 3.3 Dependence on molecular configuration for eight-unit porphyrin tape

**Figure 5:**
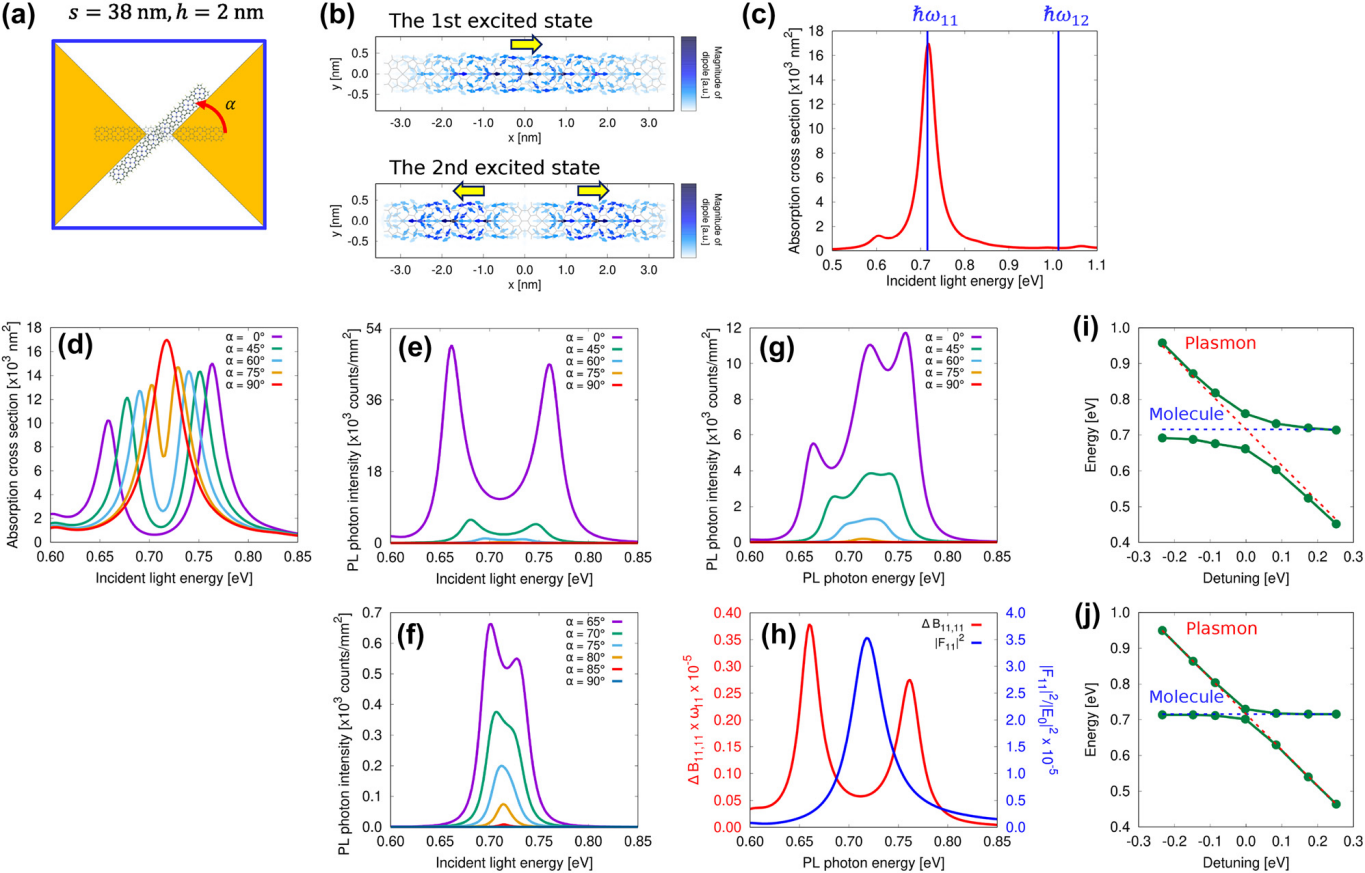
Spectra for a single eight-unit porphyrin tape. (a) Schematic representation of a porphyrin tape with eight units positioned at the center of the nanogap between the nanoplates, with *s* = 38 nm and *h* = 2 nm, rotated by an angle *α*. (b) Distribution of the transition dipole in the first excited state (allowed transition) and the second excited state (forbidden transition), with the net direction of the transition dipole moment within the molecule indicated by yellow arrows. (c) Absorption spectrum of the nanoplate dimer for *s* = 38 nm and *h* = 2 nm, with blue lines representing the resonance energy of the eight-unit porphyrin tape. (d) Absorption spectrum of the coupled system and (e) excitation spectrum dependent on *α*. (f) Detailed view of the excitation spectrum for *α* ranging from 65° to 90°. (g) PL spectrum dependence on *α*. (h) Spectra of |*F*_11_(*ω*_out_)|^2^ (blue line) and $\Delta {\bar{B}}_{11,11}\left(\omega_{out}\right)$ (red line) for *α* = 0. (i) Polariton states resulting from strong coupling between the plasmon and the exciton of the molecule, with red and blue lines indicating the plasmon and molecular resonances, respectively. (j) Similar dispersion derived from the absorption spectrum for *α* = 75°.

Here, we discuss how the spectra for absorption, excitation, and PL vary under strong coupling depending on the angle of the eight-unit porphyrin tape molecule. Figure 5(a) depicts an eight-unit porphyrin tape positioned at the center of the nanogap between nanoplates, with *s* = 38 nm and *h* = 2 nm, rotated by an angle *α*. The calculations for the eight-unit system use the same nanoplate size as in the previous model. Figure 5(b) illustrates the transition dipole distribution in the first and second excited states of the porphyrin tape. In the first excited state, corresponding to the allowed transition, the excited molecule exhibits a dipole moment in the *x*-direction. In contrast, the second excited state, corresponding to the forbidden transition, results in a nullified net dipole moment, as indicated by the yellow arrows. Figure 5(c) presents the absorption spectrum of the metallic nanoplates without the molecule, with *s* = 38 nm and *h* = 2 nm. The blue lines indicate the molecular resonance energy of the eight-unit porphyrin tape. The eight-unit porphyrin tape exhibits a sufficiently large transition dipole moment, enabling the formation of a strong coupling state. Additionally, it demonstrates strong anisotropy, providing further degrees of freedom in its orientation.

In our theoretical framework, the coupling strength is determined by the geometric configuration of the molecule and metallic nanostructure. A change in the molecular angle corresponds to a continuous change in coupling strength [65]. Figure 5(d) presents the absorption spectrum of the combined metallic nanostructure and molecule. As *α* increases, the separation between the splitting peaks decreases due to diminishing coupling between the nanocavity and molecule. For *α* = 90°, the absorption spectrum exhibits the same characteristics as the spectrum of the metallic nanoplates without the molecule, as shown in Figure 5(c). This occurs because the excitation of the molecule is nullified by the symmetry of the molecule and nanocavity. Figure 5(e) shows the excitation spectrum derived from the PL intensity at *ℏω*_out_ = 0.6557 eV. For *α* = 0, the excitation spectrum exhibits clear RS, indicating strong coupling between the nanocavity and molecule. As *α* increases, the PL intensity and the separation between the splitting peaks decrease, eventually vanishing for *α* = 90°. Figure 5(f) illustrates the dependence of the excitation spectrum on *α*, ranging from *α* = 65° to 90°. When *α* reaches 75°, the splitting peaks become indiscernible, and the coupled system transitions to a weak coupling state. Comparing the excitation and absorption spectra for *α* = 75°, the excitation spectrum displays a single peak, whereas the absorption spectrum exhibits an RS-like structure, as shown in Figure 5(d).

Figure 5(g) shows the PL spectrum for incident light at *ℏω*_in_ = 0.7957 eV. In the strong coupling state, three splitting peaks are observed in the PL spectrum, in contrast to the peaks in the excitation spectrum. For further analysis, $|F_{11}\left(r_{\text{d}},\omega_{out}\right){|}^{2}$ and $\Delta {\bar{B}}_{11,11}\left(\omega_{out}\right)$ from the PL formula (Eq. (10)) are plotted for *α* = 0°, as shown in Figure 5(h). These components significantly contribute to the PL spectrum of the first excited state. $|F_{11}\left(r_{\text{d}},\omega_{out}\right){|}^{2}$ exhibits a plasmon resonance peak, whereas $\Delta {\bar{B}}_{11,11}\left(\omega_{out}\right)$ displays two splitting peaks analogous to those in the excitation spectrum. The PL spectral features under strong coupling are obtained by multiplying these components. These results indicate that the PL spectrum comprises two peaks due to RS and an additional peak resulting from PL enhancement by the plasmonic nanocavity. In contrast, the absorption process does not enhance emission in the frequency region between the two Rabi-split peaks, and no additional peak appears in the excitation spectrum.

Subsequently, we fixed *α* = 0 and varied the side length of the nanoplates, *s*, from 32 nm to 44 nm in increments of 2 nm. Detuning is defined as *δ* = *ℏω*_mol_ − *ℏω*_pla_, where *ℏω*_mol_ and *ℏω*_pla_ represent the molecular resonance of the 1st excited state and the plasmon resonance of the nanoplate dimer, respectively. Using this methodology, we obtained the polariton states resulting from strong coupling between the plasmon and molecular exciton modes, as illustrated in Figure 5(i), where the points were obtained from the peaks in the excitation spectrum for *α* = 0. The energy gap between the anticrossing significantly exceeds the damping of the cavity, approximately 40 meV, as determined from the absorption spectrum in Figure 5(c), and that of the molecule, which is 10 meV. Figure 5(j) shows the results when the absorption peaks for *α* = 75° are considered. It is important to note that in this ET case, a dispersion profile similar to that of the RS case may still be obtained, necessitating caution in interpretation.

**Figure 6:**
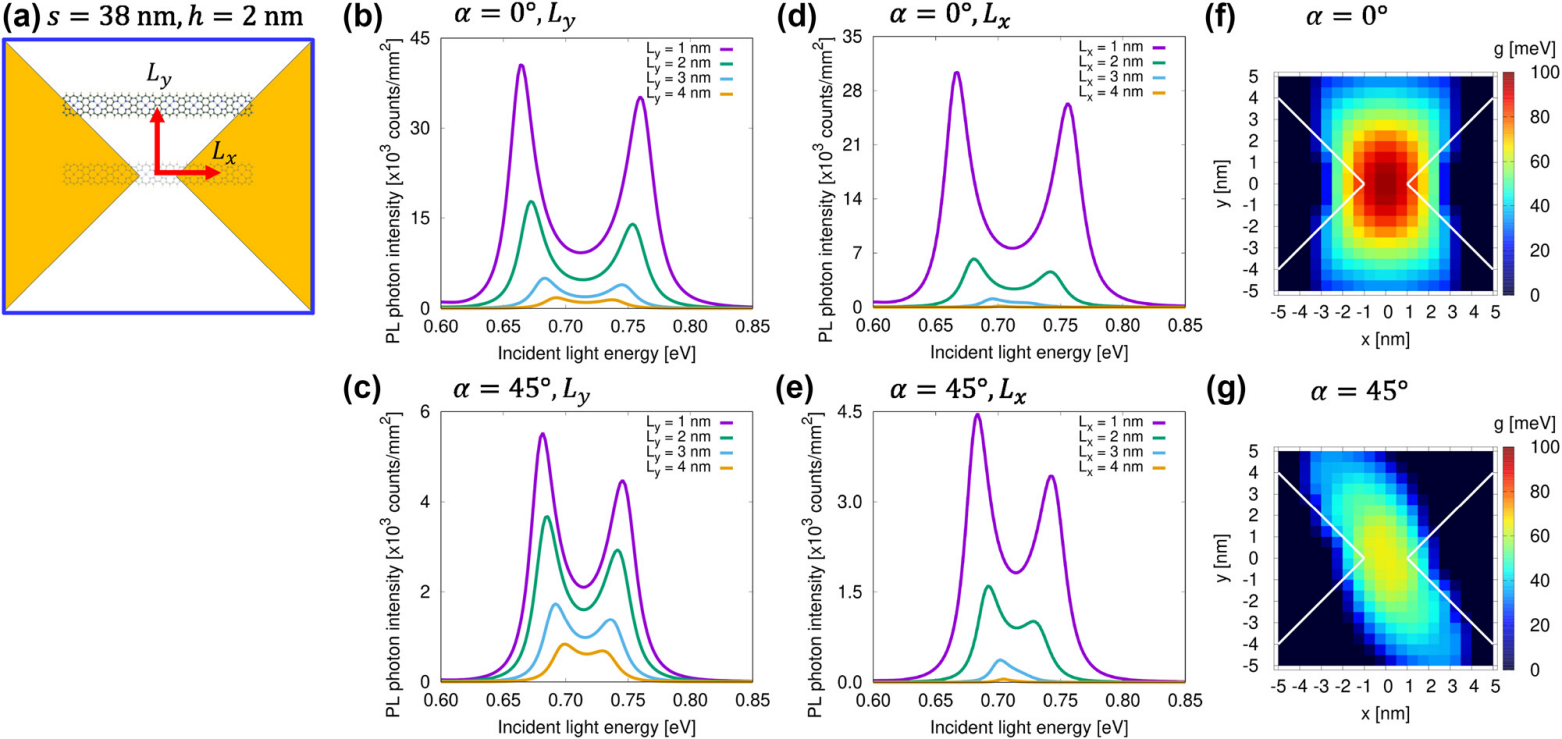
Excitation spectra and coupling strength mapping as a function of the eight-unit tape position. (a) Schematic of a molecule shifted from the center of the nanogap, with the molecular angle *α* fixed. The excitation spectrum as a function of *L*_*y*_ for (b) *α* = 0° and (c) *α* = 45°. Mapping of the coupling strength *g* as a function of the molecular position for (f) *α* = 0° and (g) *α* = 45°, with white lines indicating the boundaries of the metallic nanostructures.

We subsequently discuss the strong coupling state depending on the molecular position and rotation angle. Figure 6(a) shows a schematic representation of the molecule displaced from the center of the nanogap, with the molecular rotation angle *α* fixed. The displacement length is defined as *L*_*x*_ along the *x*-axis and *L*_*y*_ along the *y*-axis. Figure 6(b) and (c) illustrates the dependence of the excitation spectrum on *L*_*y*_ for *α* = 0° and *α* = 45°, respectively. Similarly, Figure 6(d) and (e) depicts the dependence of the excitation spectrum on *L*_*x*_ for *α* = 0° and *α* = 45°, respectively. When the molecule is displaced along the *y*-axis, the enhancement and coupling strength diminish; however, splitting in the spectrum remains observable, even for *α* = 45°. In contrast, when the molecule is displaced along the *x*-axis, a more pronounced decrease in the enhancement and coupling strength occurs compared to displacement along the *y*-axis. By analyzing the variation in molecular position and angle, we estimate the spatial region where the strong coupling state can be observed. The coupling strength *g* is derived from the separation between the upper and lower peak energies in the excitation spectrum. Figure 6(f) and (g) presents the distribution of the coupling strength *g* as a function of the molecular position for *α* = 0° and *α* = 45°, respectively. Strong coupling occurs in the region where *g* exceeds the system’s damping rate of approximately 40 meV, indicating that the strong coupling state can be clearly observed in the vicinity of the nanogap. For *α* = 0°, strong coupling is distributed within *L*_*x*_ ≤ 2.5 nm and *L*_*y*_ ≤ 4 nm. Even when the molecule is rotated to *α* = 45°, strong coupling is distributed within *L*_*x*_ ≤ 2 nm and *L*_*y*_ ≤ 3 nm, extending diagonally to reflect the molecular orientation. These analyses provide valuable insights into the quantum state and coupling behavior of a molecule as functions of its position and angle.

### 3.4 Optical forbidden transition

**Figure 7:**
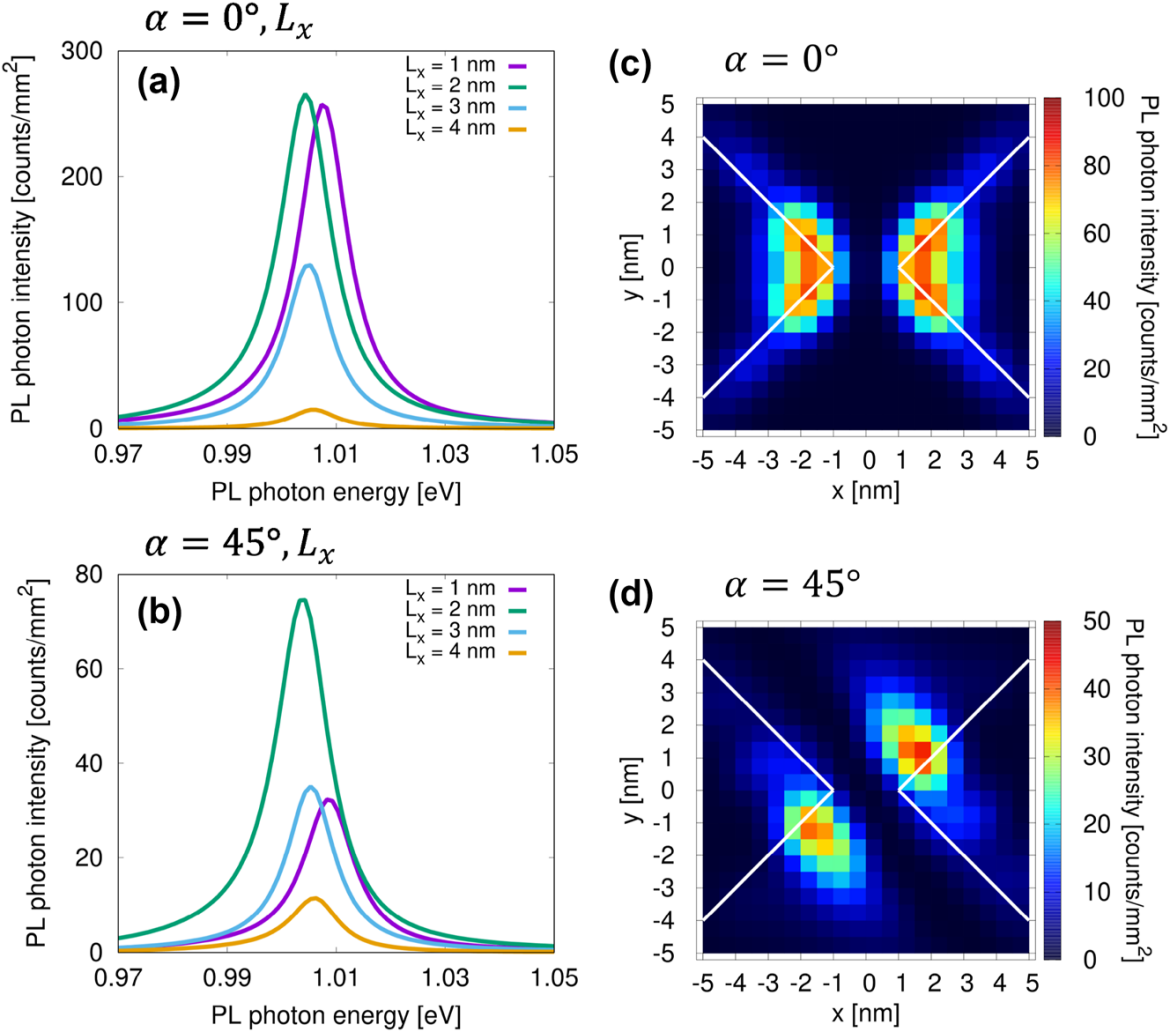
PL spectra and mapping for optically forbidden transtion. (a) PL spectrum as a function of *L*_*x*_ for *α* = 0°. (b) PL spectrum as a function of *L*_*x*_ for *α* = 45°. (c) Mapping of the averaged PL intensity over the PL photon energy as a function of molecular position for *α* = 0°. (d) Mapping of the averaged PL intensity over the PL photon energy as a function of molecular position for *α* = 45°.

Finally, to highlight the effect of the nonlocal response, we discuss the coupling between the nanocavity and the molecule in the second excited state, corresponding to the optically forbidden transition. We investigated the relationship between PL intensity and the molecular position in proximity to the nanogap. Figure 7(a) and (b) shows the PL spectrum as a function of *L*_*x*_ (defined in Figure 6(a)) for *α* = 0° and *α* = 45°, respectively. The incident light energy was set to *ℏω*_in_ = 1.0123 eV, matching the molecular resonance of the second excited state. PL intensity maps within the range of 0.9723–1.0323 eV were calculated in steps of 5 meV and averaged. The averaged PL intensity maps for *α* = 0° and *α* = 45° are shown in Figure 7(c) and (d), respectively. When the molecule is shifted along the *x*-axis, variations in the PL intensity and peak shift as a function of *L*_*x*_ are observed, attributable to localized excitation within the molecule induced by the nanogap. Interestingly, PL intensity is detectable at positions displaced from the nanogap center, while no PL is observed at *L*_*x*_ = 0. PL occurs only when one of the oppositely oriented transition dipole moments, distributed on both sides of the molecule, is positioned at the nanogap center. When the center of the molecule is aligned with the nanogap, the interaction described by Eq. (2) becomes zero due to the symmetry between the transition dipole moment and the light field in the nanogap, resulting in no PL. This phenomenon cannot be accurately evaluated if the molecule is represented as a single dipole under the long-wavelength approximation. While using two dipoles can approximate the effect, it remains insufficient when the electric field varies drastically within the nanogap. To accurately capture this behavior, it is crucial to consider the geometric relationship between the metallic nanostructure and the molecule, particularly the correlation between the distribution of the molecule’s transition dipole moment and the localized electric field in the nanogap. The nonlocal response theory enables the natural evaluation of signals from optically forbidden transitions, facilitating the analysis of the coupling state between the nanocavity and molecule for excited states corresponding to forbidden transitions.

## 4 Conclusions

This study presents a theoretical framework based on nonlocal response theory, enabling a comprehensive analysis of the photoluminescence (PL) spectrum in strongly coupled systems comprising a single molecule and a plasmonic nanocavity. The interaction between the enhanced electric field, generated by the localized surface plasmon resonance of arbitrarily structured metallic nanostructures, and molecular polarizations derived from specific molecular orbitals is treated self-consistently. By setting the Green’s function frequency to the actual PL frequency rather than fixing it to the molecular resonance, our framework accurately describes PL from states significantly shifted from the original molecular frequency, facilitating the evaluation of PL spectra in strongly coupled nanocavity systems.

The transition from weak to strong coupling regimes was effectively demonstrated by increasing the number of units in porphyrin tapes. The excitation spectrum provided detailed insights into the quantum state of the molecule coupled with the plasmon, as compared to the absorption spectrum. Distinct split peaks were observed across the excitation, PL, and absorption spectra. For example, with an eight-unit porphyrin tape exhibiting a large transition dipole moment, clear Rabi splitting (RS) was observed in the excitation spectra, while the PL spectrum revealed three peaks: two associated with RS and one due to plasmonic enhancement within the nanocavity. The coupling strength and spectral features were found to strongly depend on the molecular position and orientation relative to the nanocavity, with strong coupling achievable within a few nanometers of the gap center.

This theoretical framework provides valuable insights into quantum states involving multipole excitation in molecules, enabled by the plasmonic nanocavity through nonlocal treatment. The results present a realistic approach that incorporates the complete geometric relationship between the nanostructure and molecular orbitals, ensuring accurate modeling of strongly coupled systems. This work advances the theoretical understanding of molecule-plasmon interactions and offers a robust tool for designing and optimizing strongly coupled systems in nanophotonics and quantum optics.

## Supplementary Material

Supplementary Material Details
