# Supplementary material for: Enhanced photoluminescence of strongly coupled single molecule-plasmonic nanocavity: analysis of spectral modifications using nonlocal response theory
Source: Nanophotonics. 2025 Feb 17;14(8):1157–69. doi: 10.1515/nanoph-2024-0580 (PMC12019952; doi:10.1515/nanoph-2024-0580)
Supplement: Supplementary file 1 — Supplementary Material Details [file j_nanoph-2024-0580_suppl_001.pdf]

Enhanced photoluminescence of strongly coupled single molecule-plasmonic nanocavity: Analysis of spectral modifications using nonlocal response theory

Yoshitsugu Tomoshige,† Mamoru Tamura,*,†,‡ Tomohiro Yokoyama,† and Hajime Ishihara*,†,¶

†*Department of Materials Engineering Science, Osaka University, 1-3 Machikaneyama-cho, Toyonaka, Osaka 560-8531, Japan*

‡*RILACS, Osaka Metropolitan University, 1-1 Gakuen-cho, Naka-ku, Sakai, Osaka 599-8231, Japan*

¶*Ritsumeikan Semiconductor Application research center (RISA), Ritsumeikan University, Kusatsu, Shiga 525-8577, Japan*

E-mail: mtamura@mp.es.osaka-u.ac.jp; ishi@mp.es.osaka-u.ac.jp

S1. Theoretical framework

S1.1. Hamiltonian

The Hamiltonian of the plasmonic nanocavity-molecule coupled system is described as

$$\hat{\mathcal{H}} = \hat{\mathcal{H}}_{\text{mol}} + \hat{\mathcal{H}}_{\text{rad}} + \hat{\mathcal{H}}_{\text{int}}. \tag{1}$$

The Hamiltonian of the single molecule is described as

$$\hat{\mathcal{H}}_{\text{mol}} = \sum_{k,l} \hbar\omega_{kl}\hat{b}^{\dagger}_{kl}\hat{b}_{kl}, \tag{2}$$

where ω_{kl} is the transition frequency between the molecule orbitals ϕ_k and ψ_l, and $\hat{b}_{kl}$ ($\hat{b}^{\dagger}_{kl}$) is bosonic annihilation (creation) operator of the one-electron excitation between ϕ_k and ψ_l. The Hamiltonian of the radiation field is expressed as

$$\hat{\mathcal{H}}_{\text{rad}} = \sum_{\eta} \hbar\Omega_{\eta}\hat{a}^{\dagger}_{\eta}\hat{a}_{\eta}, \tag{3}$$

where $\hat{a}_{\eta}$ ($\hat{a}^{\dagger}_{\eta}$) is the bosonic annihilation (creation) operator of the η th photon with eigenfrequency Ω_{η}. The Hamiltonian of the interaction between the molecule and the electric field is

$$\hat{\mathcal{H}}_{\text{int}} = -\int \mathrm{d}\mathbf{r}\,\hat{\mathbf{P}}_{\text{mol}}(\mathbf{r})\cdot\hat{\mathbf{E}}(\mathbf{r}). \tag{4}$$

$\hat{\mathbf{P}}_{\text{mol}}(\mathbf{r})$ is the molecular polarization operator expressed as

$$\hat{\mathbf{P}}_{\text{mol}}(\mathbf{r}) = \sum_{k,l}\left[\boldsymbol{\mathcal{P}}_{kl}(\mathbf{r})\hat{b}_{kl} + \boldsymbol{\mathcal{P}}^{*}_{kl}(\mathbf{r})\hat{b}^{\dagger}_{kl}\right], \tag{5}$$

where $\boldsymbol{\mathcal{P}}_{kl}(\mathbf{r})$ denotes transition dipole. The distribution of the transition dipole is described using the local dipole moment in a small unit volume V_j around the position $\mathbf{r}_j$ expressed as[1]

$$\delta\mu^{j}_{kl} = \frac{ie}{\omega_{kl}}\int_{V_j}\mathrm{d}\mathbf{r}\,\mathbf{j}_{kl}(\mathbf{r}), \tag{6}$$

$\mathbf{j}_{kl}(\mathbf{r})$ is the transition current density described as

$$\mathbf{j}_{kl}(\mathbf{r}) = -\frac{i\hbar}{2m_e}[\psi_l^*(\mathbf{r})\nabla\phi_k(\mathbf{r}) - \phi_k^*(\mathbf{r})\nabla\psi_l(\mathbf{r})], \tag{7}$$

where m_e is the mass of the electron.

The electric field operator $\hat{\mathbf{E}}(\mathbf{r})$ is expressed as

$$\hat{\mathbf{E}}(\mathbf{r}) = \sum_\eta \sqrt{\frac{\hbar\Omega_\eta}{2\varepsilon_0}} \left[i\boldsymbol{\mathcal{E}}_\eta(\mathbf{r})\hat{a}_\eta - i\boldsymbol{\mathcal{E}}_\eta^*(\mathbf{r})\hat{a}_\eta^\dagger\right], \tag{8}$$

where $\boldsymbol{\mathcal{E}}_\eta(\mathbf{r})$ represents an eigenfunction that satisfies Maxwell's equation in the absence of a source.

S1.2. Derivation of the electric field expression

Using Hamiltonian of Eq. (1) and from the Heisenberg equation, the equation of motion for photons is given by:

$$\frac{\partial}{\partial t}\hat{a}_\eta(t) = \frac{1}{i\hbar}\left[\hat{a}_\eta(t), \hat{H}\right] \tag{9}$$

$$= -i\Omega_\eta\hat{a}_\eta(t) + \frac{\alpha_\eta}{\hbar}\int \mathrm{d}\mathbf{r} \sum_{m,n} \boldsymbol{\mathcal{E}}_\eta^*(\mathbf{r}) \cdot \boldsymbol{\mathcal{P}}_{mn}(\mathbf{r})\hat{b}_{mn}(t). \tag{10}$$

For the time range $t_0 < t < t_1$, the range $t_0 < t$ is defined as the "input," and $t < t_1$ is defined as the "output."

The solution to Eq. (10) is derived in the input range $t_0 < t$.

$$\hat{a}_\eta(t) = -e^{-i\Omega_\eta(t-t_0)}\hat{a}_\eta(t_0) + \frac{\alpha_\eta}{\hbar}\sum_{m,n}\int \mathrm{d}\mathbf{r}\, \boldsymbol{\mathcal{E}}_\eta^*(\mathbf{r}) \cdot \boldsymbol{\mathcal{P}}_{mn}(\mathbf{r}) \int_{t_0}^{t} \mathrm{d}t'\, e^{-i\Omega_\eta(t-t')}\hat{b}_{mn}(t'). \tag{11}$$

From the Heisenberg equation, the equation of motion for excitons is given by:

$$\begin{aligned}\frac{\partial}{\partial t}\hat{b}_{kl}(t) &= \frac{1}{i\hbar}\left[\hat{b}_{kl}(t), \hat{H}\right] \\ &= -i\omega_{kl}\hat{b}_{kl}(t) - \frac{1}{\hbar}\int \mathrm{d}\mathbf{r}\sum_{\eta}\alpha_{\eta}\boldsymbol{\mathcal{P}}^{*}_{kl}(\mathbf{r})\cdot\boldsymbol{\mathcal{E}}_{\eta}(\mathbf{r})\hat{a}_{\eta}(t).\end{aligned} \tag{12}$$

Substitute the equation for $\hat{a}_{\eta}(t)$ in Eq. (11) into the equation of motion for $\hat{b}_{kl}(t)$ in Eq. (12) for $\hat{a}_{\eta}(t)$:

$$\begin{aligned}\frac{\partial}{\partial t}\hat{b}_{kl}(t) = -i\omega_{kl}\hat{b}_{kl}(t) &- \frac{1}{\hbar}\int d\mathbf{r}\sum_{\eta}\alpha_{\eta}\boldsymbol{\mathcal{P}}^{*}_{kl}(\mathbf{r})\cdot\boldsymbol{\mathcal{E}}(\mathbf{r})e^{-i\Omega_{\eta}(t-t_0)}\hat{a}_{\eta}(t_0) \\ &+ \frac{1}{\hbar}\sum_{m,n}\int_{t_0}^{t}\mathrm{d}t'\int d\mathbf{r}\int d\mathbf{r}'\boldsymbol{\mathcal{P}}^{*}_{kl}(\mathbf{r})\sum_{\eta}\frac{i\Omega_{\eta}\boldsymbol{\mathcal{E}}_{\eta}(\mathbf{r})\boldsymbol{\mathcal{E}}^{*}_{\eta}(\mathbf{r}')}{2\varepsilon_0}e^{-i\Omega_{\eta}(t-t_0)}\boldsymbol{\mathcal{P}}_{mn}(\mathbf{r})\hat{b}_{mn}(t').\end{aligned} \tag{13}$$

The Green's function $\mathbf{G}(\mathbf{r},\mathbf{r}',t-t')$, the incident field $\hat{\mathbf{E}}_{\text{in}}(\mathbf{r},t)$, and the induced field $\hat{\mathbf{E}}^{<}_{\text{ind}}(\mathbf{r},t)$ are defined as:

$$\mathbf{G}(\mathbf{r},\mathbf{r}',t-t') \equiv \sum_{\eta}\frac{i\Omega_{\eta}\boldsymbol{\mathcal{E}}_{\eta}(\mathbf{r})\boldsymbol{\mathcal{E}}^{*}_{\eta}(\mathbf{r}')}{2\varepsilon_0}e^{-i\Omega_{\eta}(t-t')}, \tag{14a}$$

$$\hat{\mathbf{E}}_{\text{in}}(\mathbf{r},t) \equiv \sum_{\eta} i\alpha_{\eta}\boldsymbol{\mathcal{E}}_{\eta}(\mathbf{r})e^{-i\Omega_{\eta}(t-t_0)}\hat{a}_{\eta}(t_0), \tag{14b}$$

$$\hat{\mathbf{E}}^{<}_{\text{ind}}(\mathbf{r},t) \equiv \sum_{m,n}\int_{t_0}^{t}\mathrm{d}t'\int d\mathbf{r}\mathbf{G}(\mathbf{r},\mathbf{r}',t-t')\boldsymbol{\mathcal{P}}_{mn}(\mathbf{r}')\hat{b}_{mn}(t'). \tag{14c}$$

Therefore, The equation of motion for the exciton at $t_0 < t$ is given by:

$$\frac{\partial}{\partial t}\hat{b}_{kl}(t) = -i\omega_{kl}\hat{b}_{kl}(t) + \frac{i}{\hbar}\int d\mathbf{r}\boldsymbol{\mathcal{P}}^{*}_{kl}(\mathbf{r})\cdot\hat{\mathbf{E}}_{\text{in}}(\mathbf{r},t) + \frac{i}{\hbar}\int d\mathbf{r}\boldsymbol{\mathcal{P}}^{*}_{kl}(\mathbf{r})\cdot\mathbf{E}^{<}_{\text{ind}}(\mathbf{r},t). \tag{15}$$

The solution of Eq. (10) for $t < t_1$ is given by:

$$\hat{a}_\eta(t) = e^{-\Omega_\eta(t-t_1)}\hat{a}_\eta(t_1) - \frac{\alpha_\eta}{\hbar}\sum_{m,n}\int d\mathbf{r}\boldsymbol{\mathcal{E}}^*_\eta(\mathbf{r})\cdot\boldsymbol{\mathcal{P}}_{mn}(\mathbf{r})\int_t^{t_1}\mathrm{d}t'\, e^{-i\Omega_\eta(t-t')}\hat{b}_{mn}(t'). \tag{16}$$

Substitute Eq. (16) into Eq. (12):

$$\begin{aligned}\frac{\partial}{\partial t}\hat{b}_{kl}(t) = -i\omega_{kl}\hat{b}_{kl}(t) - \frac{1}{\hbar}\int d\mathbf{r}\sum_\eta \alpha_\eta\boldsymbol{\mathcal{P}}^*_{kl}(\mathbf{r})\cdot\boldsymbol{\mathcal{E}}_\eta(\mathbf{r})\Big[e^{-\Omega_\eta(t-t_1)}\hat{a}_\eta(t_1)\\ -\frac{\alpha_\eta}{\hbar}\sum_{m,n}\int d\mathbf{r}'\boldsymbol{\mathcal{E}}^*_\eta(\mathbf{r}')\cdot\boldsymbol{\mathcal{P}}_{mn}(\mathbf{r}')\int_t^{t_1}\mathrm{d}t'\, e^{-i\Omega_\eta(t-t')}\hat{b}_{mn}(t')\Big]\end{aligned} \tag{17}$$

$$\begin{aligned}= -i\omega_{kl}\hat{b}_{kl}(t) - \frac{1}{\hbar}\int d\mathbf{r}\sum_\eta \alpha_\eta\boldsymbol{\mathcal{P}}^*_{kl}(\mathbf{r})\cdot\boldsymbol{\mathcal{E}}_\eta(\mathbf{r})e^{-i\Omega_\eta(t-t_1)}\hat{a}_\eta(t_1)\\ -\frac{i}{\hbar}\sum_\eta\int_t^{t_1}\mathrm{d}t'\int d\mathbf{r}\int d\mathbf{r}'\boldsymbol{\mathcal{P}}^*_{kl}(\mathbf{r})\sum_\eta\frac{i\Omega_\eta\boldsymbol{\mathcal{E}}_\eta(\mathbf{r})\boldsymbol{\mathcal{E}}^*_\eta(\mathbf{r}')}{2\varepsilon_0}e^{-\Omega_\eta(t-t')}\boldsymbol{\mathcal{P}}_{mn}(\mathbf{r}')\hat{b}_{mn}(t').\end{aligned} \tag{18}$$

The Green's function $\mathbf{G}(\mathbf{r},\mathbf{r}',t-t')$, the output field $\mathbf{E}_{\text{out}}(\mathbf{r},t)$, and the induced field $\mathbf{E}^{>}_{\text{ind}}(\mathbf{r},t)$ are given by:

$$\mathbf{G}(\mathbf{r},\mathbf{r}',t-t') \equiv \sum_\eta\frac{i\Omega_\eta\boldsymbol{\mathcal{E}}_\eta(\mathbf{r})\boldsymbol{\mathcal{E}}^*_\eta(\mathbf{r}')}{2\varepsilon_0}e^{-i\Omega_\eta(t-t')}, \tag{19a}$$

$$\hat{\mathbf{E}}_{\text{out}}(\mathbf{r},t) \equiv \sum_\eta i\alpha_\eta\boldsymbol{\mathcal{E}}_\eta(\mathbf{r})e^{-i\Omega_\eta(t-t_1)}\hat{a}_\eta(t_1), \tag{19b}$$

$$\hat{\mathbf{E}}^{>}_{\text{ind}}(\mathbf{r},t) \equiv \sum_{m,n}\int_t^{t_1}\mathrm{d}t'\int d\mathbf{r}\mathbf{G}(\mathbf{r},\mathbf{r}',t-t')\boldsymbol{\mathcal{P}}_{mn}(\mathbf{r}')\hat{b}_{mn}(t'). \tag{19c}$$

The equation of motion for the exciton for $t < t_1$ is:

$$\frac{\partial}{\partial t}\hat{b}_{kl}(t) = -i\omega_{kl}\hat{b}_{kl}(t) + \frac{i}{\hbar}\int d\mathbf{r}\boldsymbol{\mathcal{P}}^*_{kl}(\mathbf{r})\cdot\mathbf{E}_{\text{out}}(\mathbf{r},t) - \frac{i}{\hbar}\int d\mathbf{r}\boldsymbol{\mathcal{P}}^*_{kl}(\mathbf{r})\cdot\mathbf{E}^{>}_{\text{ind}}(\mathbf{r},t). \tag{20}$$

Here, the induced field is given by:

$$\hat{\mathbf{E}}_{\text{ind}}(\mathbf{r},t) = \hat{\mathbf{E}}^{<}_{\text{ind}}(\mathbf{r},t) + \hat{\mathbf{E}}^{>}_{\text{ind}}(\mathbf{r},t). \tag{21}$$

Comparing Eq. (15) and Eq. (20) gives:

$$\int d\mathbf{r}\boldsymbol{\mathcal{P}}_{kl}(\mathbf{r}) \cdot (\hat{\mathbf{E}}_{\text{out}}(\mathbf{r},t) - \hat{\mathbf{E}}_{\text{in}}(\mathbf{r},t) - \hat{\mathbf{E}}_{\text{ind}}(\mathbf{r},t)) = 0. \tag{22}$$

Thus, we obtain the following relationship[2] :

$$\hat{\mathbf{E}}_{\text{out}}(\mathbf{r},t) = \hat{\mathbf{E}}_{\text{in}}(\mathbf{r},t) + \hat{\mathbf{E}}_{\text{ind}}(\mathbf{r},t). \tag{23}$$

S1.3. Self-consistent equations

The density matrix of the entire system obeys the following master equation , which can be found on page 16 of the textbook by Carmichael in 1998[3]:

$$\frac{\partial}{\partial t}\hat{\rho}(t) = \frac{1}{i\hbar}\left[\hat{H}, \rho(t)\right] + \hat{\mathcal{L}}_{\text{damp}}\hat{\rho}(t) + \hat{\mathcal{L}}_{\text{phase}}\hat{\rho}(t). \tag{24}$$

$\hat{\mathcal{L}}_{\text{damp}}\hat{\rho}(t)$ is the nonradiative damping term described by

$$\hat{\mathcal{L}}_{\text{damp}}\hat{\rho}(t) \equiv \sum_{m,n}\frac{\gamma}{2}\left[2\hat{b}_{mn}\hat{\rho}(t)\hat{b}^{\dagger}_{mn} - \left\{\hat{b}^{\dagger}_{mn}\hat{b}_{mn}, \rho(t)\right\}\right]. \tag{25}$$

$\hat{\mathcal{L}}_{\text{phase}}\hat{\rho}(t)$ denotes the dephasing term described by

$$\hat{\mathcal{L}}_{\text{phase}}\hat{\rho}(t) \equiv \sum_{m,n}\frac{\Gamma}{2}\left[\left[\hat{b}^{\dagger}_{mn}\hat{b}_{mn}, \hat{\rho}(t)\right], \hat{b}^{\dagger}_{mn}\hat{b}_{mn}\right]. \tag{26}$$

From Eq. (24), we obtain the motion equation of the polarization

$$\frac{\partial}{\partial t}\left\langle \hat{b}_{kl}(t)\right\rangle = -\left(i\omega_{kl} + \frac{\gamma}{2} + \frac{\Gamma}{2}\right)\left\langle \hat{b}_{kl}(t)\right\rangle + \frac{i}{\hbar}\int \mathrm{d}\mathbf{r}\, \boldsymbol{\mathcal{P}}^*_{kl}(\mathbf{r}) \cdot \left\langle \hat{\mathbf{E}}_{\mathrm{out}}(\mathbf{r},t)\right\rangle, \tag{27}$$

where $\langle \hat{b}_{kl}(t)\rangle$ is defined as $\langle \hat{b}_{kl}(t)\rangle \equiv \mathrm{Tr}\left[\hat{\rho}(t)\hat{b}_{kl}\right]$. By applying Fourier transformation to both sides of Eq. (27), we obtain

$$\left\langle \hat{b}_{kl}(\omega)\right\rangle = \frac{1}{\hbar\omega_{kl} - \hbar\omega - i\hbar\gamma/2 - i\hbar\Gamma/2}\int \mathrm{d}\mathbf{r}\, \boldsymbol{\mathcal{P}}^*_{kl}(\mathbf{r}) \cdot \left\langle \hat{\mathbf{E}}_{\mathrm{out}}(\mathbf{r},\omega)\right\rangle. \tag{28}$$

Inserting Eq.(28) into Eq.(5) and employing the rotation approximation, we obtain

$$\left\langle \hat{\mathbf{P}}_{\mathrm{mol}}(\mathbf{r},\omega)\right\rangle = \varepsilon_0 \int \mathrm{d}\mathbf{r}'\, \chi_{\mathrm{mol}}(\mathbf{r},\mathbf{r}',\omega)\left\langle \hat{\mathbf{E}}_{\mathrm{out}}(\mathbf{r}',\omega)\right\rangle, \tag{29}$$

where $\chi_{\mathrm{mol}}(\mathbf{r},\mathbf{r}',\omega)$ is the nonlocal susceptibility described as

$$\chi_{\mathrm{mol}}(\mathbf{r},\mathbf{r}',\omega) = \frac{1}{\varepsilon_0}\sum_{k,l}\frac{\boldsymbol{\mathcal{P}}_{kl}(\mathbf{r})\boldsymbol{\mathcal{P}}^*_{kl}(\mathbf{r}')}{\hbar\omega_{kl} - \hbar\omega - i\hbar\gamma/2 - i\hbar\Gamma/2}. \tag{30}$$

The Fourier transform relationship is defined as follows:

$$f(t) = \frac{1}{2\pi}\int_{-\infty}^{\infty} d\omega\, e^{-i\omega t}F(\omega) \tag{31a}$$

$$F(\omega) = \int_{-\infty}^{\infty} dt\, e^{i\omega t}f(t). \tag{31b}$$

The Fourier transform relationship for the Green's function is expressed as:

$$\mathbf{G}(\mathbf{r},\mathbf{r}',t-t') = \frac{1}{2\pi}\int_{-\infty}^{\infty} d\omega\, e^{-i\omega(t-t')}\mathbf{G}(\mathbf{r},\mathbf{r}',\omega), \tag{32a}$$

$$\mathbf{G}(\mathbf{r},\mathbf{r}',\omega) = \int_{-\infty}^{\infty} dt\, e^{i\omega(t-t')}\mathbf{G}(\mathbf{r},\mathbf{r}',t-t'). \tag{32b}$$

The induced electric field is given by:

$$\langle \hat{\mathbf{E}}_{\text{ind}}(\mathbf{r},t)\rangle = \sum_{m,n}\int_{t_0}^{t_1} dt' \int d\mathbf{r}'\, \mathbf{G}(\mathbf{r},\mathbf{r}',t-t')\boldsymbol{\mathcal{P}}^{*}_{mn}(\mathbf{r}')\,\langle \hat{b}_{mn}(t')\rangle\,. \tag{33}$$

Assuming a steady state with the continuous input of incident light for a sufficiently long time, and taking the limits $t_0 \to -\infty$ and $t_1 \to \infty$, the Fourier transform of both sides of Eq. (33) is given by:

$$\int_{-\infty}^{\infty} dt\ \langle \hat{\mathbf{E}}_{\text{ind}}(\mathbf{r},t)\rangle\, e^{i\omega t} \tag{34}$$

$$= \sum_{m,n}\int d\mathbf{r}' \int_{-\infty}^{\infty} dt' e^{i\omega t'} \left[\int_{-\infty}^{\infty} dt\, e^{i\omega(t-t')}\mathbf{G}(\mathbf{r},\mathbf{r}',t-t')\right] \boldsymbol{\mathcal{P}}^{*}_{mn}(\mathbf{r}')\,\langle \hat{b}_{mn}(t')\rangle \tag{35}$$

$$= \sum_{m,n}\int d\mathbf{r}'\, \mathbf{G}(\mathbf{r},\mathbf{r}',\omega)\boldsymbol{\mathcal{P}}^{*}_{mn}(\mathbf{r}')\,\langle \hat{b}_{mn}(\omega)\rangle\,. \tag{36}$$

From the relationsip of Eq. (23), we obtain:

$$\langle \hat{\mathbf{E}}_{\text{out}}(\mathbf{r},\omega)\rangle = \langle \hat{\mathbf{E}}_{\text{in}}(\mathbf{r},\omega)\rangle + \sum_{m,n}\int \mathrm{d}\mathbf{r}'\, \mathbf{G}(\mathbf{r},\mathbf{r}',\omega)\boldsymbol{\mathcal{P}}_{mn}(\mathbf{r}')\,\langle \hat{b}_{mn}(\omega)\rangle \tag{37}$$

where $\langle \hat{\mathbf{E}}_{\text{in}}(\mathbf{r},\omega)\rangle$ represents the incident field, and $\mathbf{G}(\mathbf{r},\mathbf{r}',\omega)$ denotes the Green's function renormalized arbitrary metallic structure. The renormalized Green's function is defined by

$$\mathbf{G}(\mathbf{r},\mathbf{r}',\omega) = \mathbf{G}_0(\mathbf{r},\mathbf{r}',\omega) + \int \mathrm{d}\mathbf{r}''\, \mathbf{G}_0(\mathbf{r},\mathbf{r}'',\omega)\varepsilon_0\chi_{\text{met}}(\mathbf{r}'',\omega)\mathbf{G}(\mathbf{r}'',\mathbf{r}',\omega). \tag{38}$$

$\mathbf{G}_0(\mathbf{r},\mathbf{r}',\omega)$ represents the free-space Green's function and $\chi_{\text{met}}(\mathbf{r},\omega)$ denotes the metallic susceptibility. (See, page 518 of the textbook by Hecht, B. and Novotny, L in 2012[4])

The renormalized Green's function is calculated using discrete dipole approximation, as

follows:[5,6]

$$\mathbf{G}_{ij} = \mathbf{G}^0_{ij} + \sum_{j'} V_{j'} \mathbf{G}^0_{ij'} \varepsilon_0 \chi_{j'} \mathbf{G}_{j'j} \tag{39}$$

$$\sum_{j'} [\delta_{ij'} \mathbf{I} - \varepsilon_0 \chi_{j'} V_{j'} \mathbf{G}^0_{ij'}] \mathbf{G}_{j'j} = \mathbf{G}^0_{ij}. \tag{40}$$

$\mathbf{G}_{ij}$ denotes $\mathbf{G}(\mathbf{r_i}, \mathbf{r_j}, \omega)$, where i, j represent the indices of the cells. V_i denotes the volume of the i-th cell.

Inserting Eq.(37) into Eq.(28), we obtain the simultaneous linear equation as follows:

$$\sum_{m,n} S_{kl,mn}(\omega) \langle \hat{b}_{mn}(\omega) \rangle = F^0_{kl}(\omega), \tag{41}$$

where each term is defined as

$$S_{kl,mn}(\omega) \equiv \left(\hbar\omega_{kl} - \hbar\omega - \frac{i\hbar\gamma}{2} - \frac{i\hbar\Gamma}{2} \right) \delta_{km}\delta_{ln} - Z_{kl,mn}(\omega), \tag{42}$$

$$Z_{kl,mn}(\omega) \equiv \int \mathrm{d}\mathbf{r} \int \mathrm{d}\mathbf{r}' \, \boldsymbol{\mathcal{P}}^*_{kl}(\mathbf{r}) \mathbf{G}(\mathbf{r}, \mathbf{r}', \omega) \boldsymbol{\mathcal{P}}_{mn}(\mathbf{r}'), \tag{43}$$

$$F^0_{kl}(\omega) \equiv \int \mathrm{d}\mathbf{r} \, \boldsymbol{\mathcal{P}}^*_{kl}(\mathbf{r}) \cdot \langle \hat{\mathbf{E}}_{\mathrm{in}}(\mathbf{r}, \omega) \rangle . \tag{44}$$

The incident field is treated as classical continuous light.

$$\langle \hat{\mathbf{E}}_{\mathrm{in}}(\mathbf{r}, \omega) \rangle = \boldsymbol{\mathcal{E}}_{\mathrm{bg}}(\mathbf{r}, \omega) \delta(\omega - \omega_{\mathrm{in}}), \tag{45}$$

where $\boldsymbol{\mathcal{E}}_{\mathrm{bg}}(\mathbf{r}, \omega)$ represents the background field from the metallic structure without the molecule and ω_{in} denotes the frequency of the incident light. Solving the Eq.(41), the polarization and electric field are self-consistently determined.

S1.4. Derivation of the photoluminescence formula expression

Luminescence in a steady-state system is related to the power spectrum calculated by the Fourier transformation of the autocorrelation function, known as Wiener-Khintchine theorem.[7,8] The autocorrelation function of the output field is described by

$$\langle \Delta\hat{\mathbf{E}}^{\dagger}_{\mathrm{out}}(\mathbf{r},t)\Delta\hat{\mathbf{E}}_{\mathrm{out}}(\mathbf{r},t+\tau)\rangle = \langle \hat{\mathbf{E}}^{\dagger}_{\mathrm{out}}(\mathbf{r},t)\hat{\mathbf{E}}_{\mathrm{out}}(\mathbf{r},t+\tau)\rangle - \langle \hat{\mathbf{E}}^{\dagger}_{\mathrm{out}}(\mathbf{r},t)\rangle \, \langle \hat{\mathbf{E}}_{\mathrm{out}}(\mathbf{r},t+\tau)\rangle \, . \tag{46}$$

The photoluminescence spectrum observed at the detector position $\mathbf{r}_{\mathrm{d}}$ is calculated by

$$S_{\mathrm{inc}}(\mathbf{r}_{\mathrm{d}},\omega_{\mathrm{out}}) = \frac{1}{\pi}\,\mathrm{Re}\left[\int_{0}^{\infty} \mathrm{d}\tau \, e^{i\omega_{\mathrm{out}}\tau} \, \langle \Delta\hat{\mathbf{E}}^{\dagger}_{\mathrm{out}}(\mathbf{r}_{\mathrm{d}},t)\Delta\hat{\mathbf{E}}_{\mathrm{out}}(\mathbf{r}_{\mathrm{d}},t+\tau)\rangle\right]. \tag{47}$$

By utilizing Maxwell's electric field, as expressed in Eq. (37), we derived the following formula for the PL spectrum:

$$S_{\mathrm{inc}}(\mathbf{r}_{\mathrm{d}},\omega_{\mathrm{out}}) = \frac{2}{\pi}\,\mathrm{Re}\left[\sum_{k,l,k',l'} \boldsymbol{\mathcal{F}}^{*}_{kl}(\mathbf{r}_{\mathrm{d}},\omega_{\mathrm{out}}) \cdot \boldsymbol{\mathcal{F}}_{k'l'}(\mathbf{r}_{\mathrm{d}},\omega_{\mathrm{out}})\Delta\bar{B}_{kl,k'l'}(\omega_{\mathrm{out}})\right] . \tag{48}$$

In this context, we describe the electric field propagating to the detector position $\mathbf{r}_{\mathrm{d}}$ as

$$\boldsymbol{\mathcal{F}}_{kl}(\mathbf{r}_{\mathrm{d}},\omega) \equiv \int \mathrm{d}\mathbf{r}' \, \mathbf{G}(\mathbf{r}_{\mathrm{d}},\mathbf{r}',\omega)\boldsymbol{\mathcal{P}}_{kl}(\mathbf{r}') \tag{49}$$

Autocorrelation function $\Delta\bar{B}_{kl,k'l'}(\tau)$ with respect to delay time τ is defined as

$$\Delta\bar{B}_{kl,k'l'}(\tau) = \langle \hat{b}^{\dagger}_{kl}(t)\hat{b}_{k'l'}(t+\tau)\rangle - \langle \hat{b}^{\dagger}_{kl}(t)\rangle \, \langle \hat{b}_{k'l'}(t+\tau)\rangle \, . \tag{50}$$

Using Eq.(27) and quantum regression theorem,[3] we obtain:

$$\frac{\partial}{\partial t'}\langle\hat{b}^{\dagger}_{kl}(t)\hat{b}_{mn}(t')\rangle = -(i\omega_{mn}+\frac{\gamma}{2}+\frac{\Gamma}{2})\langle\hat{b}^{\dagger}_{kl}(t)\hat{b}_{mn}(t')\rangle \\ +\frac{i}{\hbar}\sum_{j',m',n'}\int_{-\infty}^{\infty}\mathrm{d}t''\, Z_{mn,m'n'}(t'-t'')\langle\hat{b}^{\dagger}_{kl}(t)\hat{b}_{m'n'}(t'')\rangle \\ +\frac{i}{\hbar}\langle\hat{b}^{\dagger}_{kl}(t)\rangle\int\mathrm{d}\mathbf{r}\,\boldsymbol{\mathcal{P}}^{*}_{mn}(\mathbf{r})\cdot\langle\mathbf{E}_{\mathrm{in}}(\mathbf{r},t')\rangle \tag{51}$$

Applying $\langle\hat{b}^{\dagger}_{kl}(t)\rangle$ from left side of Eq. (27), the following equation can be obtained:

$$\frac{\partial}{\partial t'}\langle\hat{b}^{\dagger}_{kl}(t)\rangle\,\langle\hat{b}_{mn}(t')\rangle = -(i\omega_{mn}+\frac{\gamma}{2}+\frac{\Gamma}{2})\langle\hat{b}^{\dagger}_{kl}(t)\rangle\,\langle\hat{b}_{mn}(t')\rangle \\ +\frac{i}{\hbar}\sum_{j',m',n'}\int_{-\infty}^{\infty}\mathrm{d}t''\, Z_{mn,m'n'}(t'-t'')\langle\hat{b}^{\dagger}_{kl}(t)\rangle\,\langle\hat{b}_{mn}(t'')\rangle \\ +\frac{i}{\hbar}\langle\hat{b}^{\dagger}_{kl}(t)\rangle\int\mathrm{d}\mathbf{r}\,\boldsymbol{\mathcal{P}}^{*}_{mn}(\mathbf{r})\cdot\langle\mathbf{E}_{\mathrm{in}}(\mathbf{r},t')\rangle \tag{52}$$

Then, using the definitions $t'-t=\tau$ and $t'-t''=\tau'$, and referring to Eq. (51) and Eq. (52), the equation of motion is derived as follows:

$$\frac{\partial}{\partial\tau}\langle\Delta\bar{B}_{kl,k'l'}(\tau)\rangle = -(i\omega_{k'l'}+\frac{\gamma}{2}+\frac{\Gamma}{2})\langle\Delta\bar{B}_{kl,k'l'}(\tau)\rangle \tag{53}$$

$$+\frac{i}{\hbar}\sum_{m,n}\int_{-\infty}^{\infty}\mathrm{d}\tau'\, Z_{k'l',mn}(\tau')\langle\Delta\bar{B}_{kl,k'l'}(\tau-\tau')\rangle\,. \tag{54}$$

Applying the Laplace transformation to both sides of Eq.(54), the following equation is obtained.

$$\int_{0}^{\infty}\mathrm{d}\tau\, e^{i\omega_{\mathrm{out}}\tau}\frac{\partial}{\partial\tau}\Delta B_{kl,k'l'}(\tau) \\ = -(i\omega_{k'l'}+\frac{\gamma}{2}+\frac{\Gamma}{2})\int_{0}^{\infty}\mathrm{d}\tau\, e^{i\omega_{\mathrm{out}}\tau}\Delta B_{kl,k'l'}(\tau) \\ +\frac{i}{\hbar}\sum_{m,n}\int_{0}^{\infty}\mathrm{d}\tau\, e^{i\omega_{\mathrm{out}}\tau}\int_{-\infty}^{\infty}\mathrm{d}t'\, Z_{k'l',mn}(\tau')\Delta B_{kl,k'l'}(\tau-\tau'). \tag{55}$$

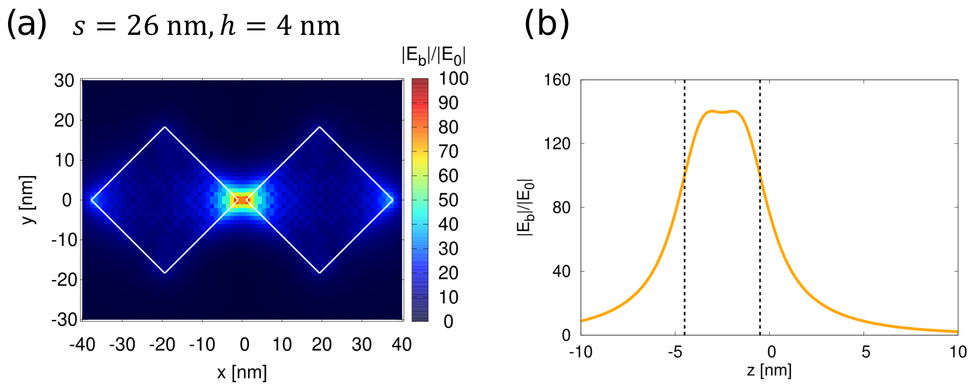

Figure 1: (a) Electric field intensity distribution of the nanoplate dimer for $s = 26$ nm and $h = 4$ nm at $z = 0$ nm, using incident light with $\hbar\omega_{\mathrm{in}} = 1.7769$ eV. (b) Electric field intensity profile along the z direction at $x = 0$ nm and $y = 0$ nm, as shown in (a). The dashed black lines indicate the positions of the nanoplate's surface and bottom.

$$\sum_{m,n}\left[\left(\hbar\omega_{k'l'} - \hbar\omega_{\mathrm{out}} - \frac{i\hbar\gamma}{2} - \frac{i\hbar\Gamma}{2}\right)\delta_{k'm}\delta_{l'n} - Z_{k'l',mn}(\omega_{\mathrm{out}})\right]\Delta\bar{B}_{kl,k'l'}(\omega_{\mathrm{out}})$$
$$= -i\hbar\Delta B_{kl,k'l'}(\tau = 0). \tag{56}$$

Right hand in Eq.(56) is obtained by

$$\Delta B_{kl,k'l'}(\tau = 0) = \langle \hat{b}^{\dagger}_{kl}\hat{b}_{k'l'}\rangle^{\mathrm{SS}} - \langle \hat{b}^{\dagger}_{kl}(t)\rangle \langle \hat{b}_{k'l'}(t)\rangle\,. \tag{57}$$

$\langle \hat{b}^{\dagger}_{kl}\hat{b}_{k'l'}\rangle^{\mathrm{SS}}$ is calculated using Eq.(24) and $\langle \hat{b}_{k'l'}(t)\rangle$ is obtained from Eq.(41) and applying Fourier transformation.

S2. Electric field profile of nanoplated dimer in height direction

Figure 1(a) illustrates the electric field intensity distribution of the nanoplate dimer for $s = 26$ nm and $h = 4$ nm at $z = 0$ nm, using incident light with $\hbar\omega_{\mathrm{in}} = 1.7769$ eV. Figure 1(b) shows the profile of the electric field intensity along the z direction at $x = 0$ nm and $y = 0$ nm, as depicted in Figure 1(a). The dashed black lines in Figure 1(b) represent the

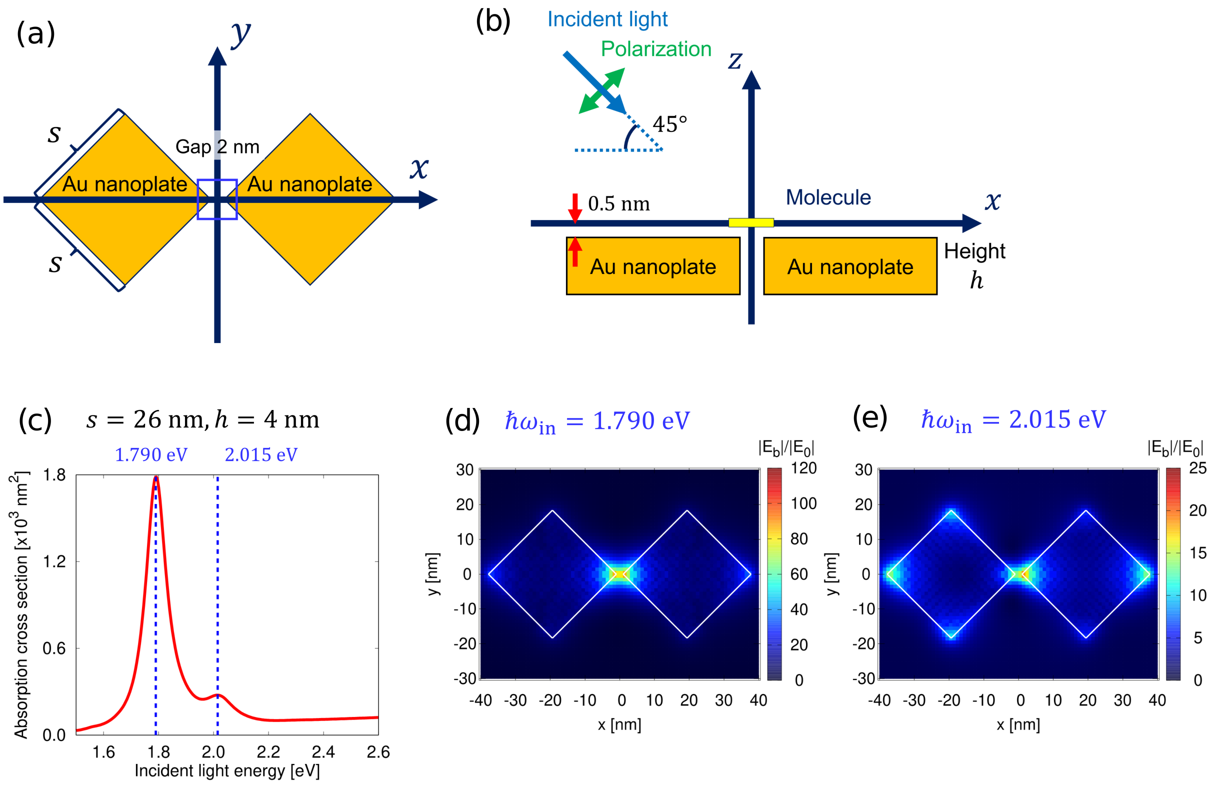

Figure 2: (a) Top view and (b) side view of the gold nanoplate dimer. (c) Absorption spectrum of the nanoplate dimer for $s = 26$ nm and $h = 4$ nm, with peak energies indicated by dashed blue lines. Electric field intensity distribution at $z = 0$ nm for $s = 26$ nm and $h = 4$ nm for (d) $\hbar\omega_{\rm in} = 1.790$ eV and (e) 2.015 eV.

positions of the nanoplate's surface and bottom along the z direction.

S3. Plasmon modes of nanoplate dimer

Figure 2(a) and (b) show the top view and side view of the gold nanoplate dimer, respectively. The side length and height of the nanoplate are denoted as s and h. Figure 2(c) presents the absorption spectrum of the nanoplate dimer for $s = 26$ nm and $h = 4$ nm, with peak energies indicated by dashed blue lines. Figures 2(d) and (e) display the electric field intensity distribution at $z = 0$ nm for $\hbar\omega_{\rm in} = 1.790$ eV and 2.015 eV, respectively.

Figure 3(a) shows the absorption spectrum of the nanoplate dimer for $s = 28$ nm and $h = 4$ nm, with peak energies indicated by dashed blue lines. Figures 3(b), (c), (d), and (e)

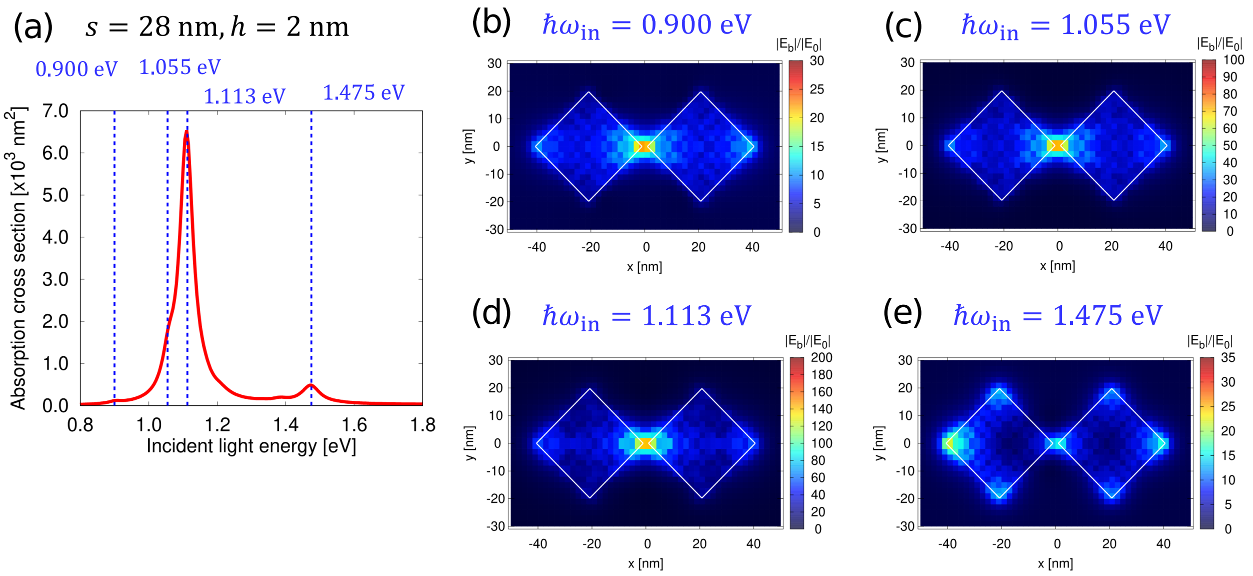

Figure 3: (a) Absorption spectrum of the nanoplate dimer for $s = 28$ nm and $h = 2$ nm, with peak energies indicated by dashed blue lines. Electric field intensity distribution at $z = 0$ nm for $s = 28$ nm and $h = 2$ nm for (b) $\hbar\omega_{\rm in} = 0.900$ eV, (c) 1.055 eV, (d) 1.113 eV, and (e) 1.475 eV.

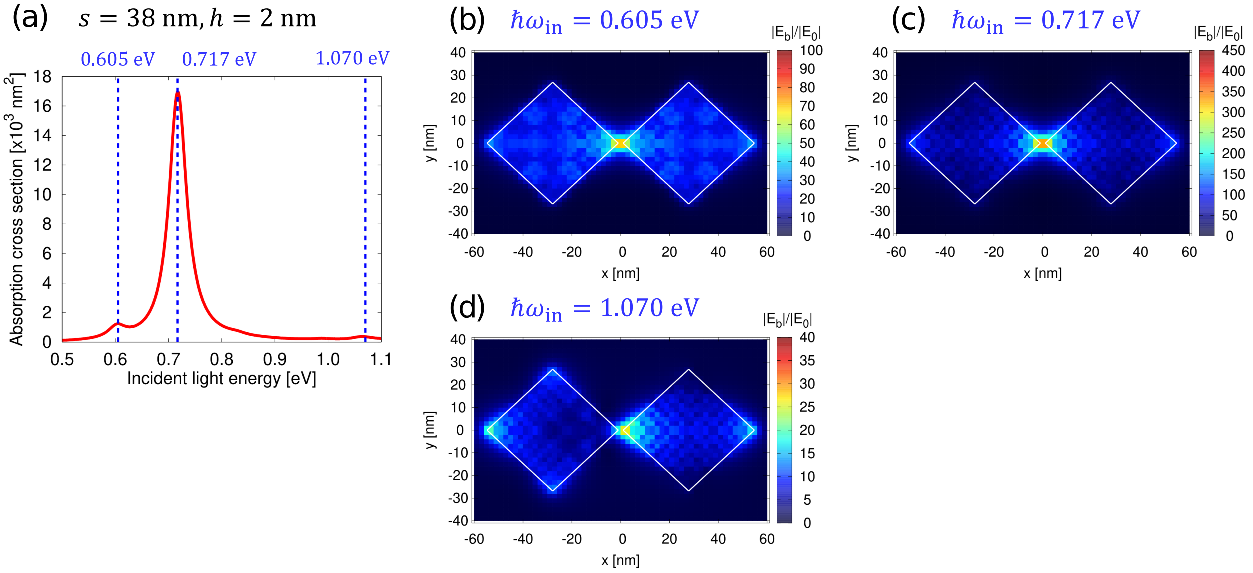

Figure 4: (a) Absorption spectrum of the nanoplate dimer for $s = 38$ nm and $h = 2$ nm, with peak energies indicated by dashed blue lines. Electric field intensity distribution at $z = 0$ nm for $s = 38$ nm and $h = 2$ nm for (b) $\hbar\omega_{\rm in} = 0.605$ eV, (c) 0.717 eV, and (d) 1.070 eV.

display the electric field intensity distribution at $z = 0$ nm for $\hbar\omega_{\rm in} = 0.900$ eV, 1.055 eV, 1.113 eV, and 1.475 eV, respectively.

Figure 4(a) shows the absorption spectrum of the nanoplate dimer for $s = 38$ nm and $h = 4$ nm, with peak energies indicated by dashed blue lines. Figures 4(b), (c), and (e) display the electric field intensity distribution at $z = 0$ nm for $\hbar\omega_{\rm in} = 0.605$ eV, 0.717 eV, and 1.070 eV, respectively.

References

(1) Zhang, Y.; Dong, Z.-C.; Aizpurua, J. Influence of the Chemical Structure on Molecular Light Emission in Strongly Localized Plasmonic Fields. *The Journal of Physical Chemistry C* **2020**, *124*, 4674–4683.

(2) Matsuda, T.; Yokoshi, N.; Ishihara, H. Upconverted photoluminescence induced by radiative coupling between excitons. *Physical Review B* **2016**, *93*, 155418.

(3) Carmichael, H. J. *Statistical methods in quantum optics 1: master equations and Fokker-Planck equations*; Springer Berlin, Heidelberg, 1998 .

(4) Hecht, B.; Novotny, L. *Principles of Nano-Optics*, 2nd ed.; Cambridge University Press: Cambridge, 2012.

(5) Purcell, E. M.; Pennypacker, C. R. Scattering and absorption of light by nonspherical dielectric grains. *Astrophysical Journal, Vol. 186, pp. 705-714 (1973)* **1973**, *186*, 705–714.

(6) Hoshina, M.; Yokoshi, N.; Okamoto, H.; Ishihara, H. Super-Resolution Trapping: A Nanoparticle Manipulation Using Nonlinear Optical Response. *ACS Photonics* **2018**, *5*, 318–323.

(7) Laussy, F. P.; del Valle, E.; Tejedor, C. Luminescence spectra of quantum dots in microcavities. I. Bosons. *Phys. Rev. B* **2009**, *79*, 235325.

(8) Mandel, L.; Wolf, E. *Optical Coherence and Quantum Optics*; Cambridge University Press, 1995.
